# Identification of *Comamonas testosteroni* as an androgen degrader in sewage

**DOI:** 10.1038/srep35386

**Published:** 2016-10-13

**Authors:** Yi-Lung Chen, Chia-Hsiang Wang, Fu-Chun Yang, Wael Ismail, Po-Hsiang Wang, Chao-Jen Shih, Yu-Ching Wu, Yin-Ru Chiang

**Affiliations:** 1 Biodiversity Research Center, Academia Sinica, Taipei, 115, Taiwan; 2 Department of Life Science, National Taiwan Normal University, Taipei, 106, Taiwan; 3 Biodiversity Program, Taiwan International Graduate Program, Academia Sinica and National Taiwan Normal University, Taipei, 115, Taiwan; 4 Environmental Biotechnology Program, Life Sciences Department, College of Graduate Studies, Arabian Gulf University, Manama, 26671, Kingdom of Bahrain; 5 Institute of Plant and Microbial Biology, Academia Sinica, Taipei, 115, Taiwan

## Abstract

Numerous studies have reported the masculinization of freshwater wildlife exposed to androgens in polluted rivers. Microbial degradation is a crucial mechanism for eliminating steroid hormones from contaminated ecosystems. The aerobic degradation of testosterone was observed in various bacterial isolates. However, the ecophysiological relevance of androgen-degrading microorganisms in the environment is unclear. Here, we investigated the biochemical mechanisms and corresponding microorganisms of androgen degradation in aerobic sewage. Sewage samples collected from the Dihua Sewage Treatment Plant (Taipei, Taiwan) were aerobically incubated with testosterone (1 mM). Androgen metabolite analysis revealed that bacteria adopt the 9, 10-*seco* pathway to degrade testosterone. A metagenomic analysis indicated the apparent enrichment of *Comamonas* spp. (mainly *C. testosteroni*) and *Pseudomonas* spp. in sewage incubated with testosterone. We used the degenerate primers derived from the *meta*-cleavage dioxygenase gene (*tesB*) of various proteobacteria to track this essential catabolic gene in the sewage. The amplified sequences showed the highest similarity (87–96%) to *tesB* of *C. testosteroni*. Using quantitative PCR, we detected a remarkable increase of the 16S rRNA and catabolic genes of *C. testosteroni* in the testosterone-treated sewage. Together, our data suggest that *C. testosteroni*, the model microorganism for aerobic testosterone degradation, plays a role in androgen biodegradation in aerobic sewage.

Steroid hormones of either natural or anthropogenic origin are ubiquitous in various environments such as manures, biosolids, soil, sediments, groundwater, and surface water^1^,^2^. These compounds typically occur at low concentrations (ng L^−1^ to μg L^−1^) in surface water^3^,^4^,^5^,^6^,^7^,^8^,^9^. However, steroid hormones have attracted increasing attention because of their ability to act as endocrine disruptors and thus adversely affect wildlife physiology and behavior, even at picomolar concentrations^10^,^11^. The masculinization of aquatic vertebrates exposed to androgens has been comprehensively reported^12^,^13^,^14^. For instance, defeminization of female fish was observed when wild fathead minnows were exposed to cattle feedlot effluent^15^.

In developed countries, sewage treatment plants are crucial for removing steroid hormones produced by humans and livestock^9^,^16^. The degradation of testosterone by microbial activity has been observed in several environmental matrices such as soil^17^, biosolids in wastewater treatment plants^18^, manure-treated soil^19^, and stream sediments^20^. Numerous studies have reported the essential role of bacterial degradation in removing these endocrine disruptors from the environment^21^,^22^,^23^. Actinobacteria and proteobacteria capable of androgen degradation have been isolated and characterized^24^,^25^,^26^,^27^. For instance, various actinobacteria, including *Rhodococcus* spp., can use androgens as the sole source of carbon and energy^26^,^27^. A betaproteobacterium, *Comamonas testosteroni*, has received special attention and its androgen catabolic intermediates and genes have been studied in detail^25^. *C. testosteroni* is frequently present in polluted environments^28^. *C. testosteroni* strains can use various hydrocarbons, including steroids (e.g., androgens and bile acids), monoaromatic compounds, acetate, and lactate, as their sole carbon sources and show resistance to heavy metals and antibiotics^29^,^30^,^31^,^32^.

As shown in Fig. 1, the aerobic degradation of testosterone by *C. testosteroni* is considered to be initiated by the dehydrogenation of the 17β-hydroxyl group to androst-4-en-3,17-dione (AD), which is then converted to androsta-1, 4-diene-3, 17-dione (ADD). The degradation of the sterane structure begins with the introduction of a hydroxyl group at C-9 of the steroid substrate^25^. The resulting intermediate is extremely unstable and undergoes simultaneous cleavage of the B-ring accompanied by aromatization of the A-ring to produce a secosteroid, 3-hydroxy-9, 10-*seco*-androsta-1, 3, 5(10)-triene-9, 17-dione (3-HSA). The further cleavage of the core ring system proceeds through hydroxylation at C-4^33^, and the A-ring is then split via TesB-mediated *meta*-cleavage. The *tesB*-disrupted mutant does not grow on testosterone, indicating that dioxygenase TesB is essential for aerobic testosterone degradation^34^. The *tesB* gene is embedded in a gene cluster of *C. testosteroni* comprising 18 androgen catabolic genes^35^. The gene cluster is widely present in androgen-degrading proteobacteria, including species within the genera *Burkholderia*, *Comamonas*, *Cupriavidus*, *Glaciecola*, *Hydrocarboniphaga*, *Marinobacterium*, *Novosphingobium*, *Pseudoalteromonas*, *Pseudomonas*, *Shewanella*, and *Sphingomonas*^25^,^36^. In addition to the well-studied 9, 10-*seco* pathway, alternative catabolic pathways of androgens have been observed in bacteria. For instance, aerobically grown *Sterolibacterium denitrificans* adopts an oxygenase-independent pathway to degrade steroid substrates^37^.

The biochemical mechanisms underlying aerobic androgen biodegradation were studied in pure cultures^25^,^33^,^34^,^38^,^39^. However, studies on the catabolic mechanisms and agents of *in situ* androgen biodegradation are lacking. It is unknown which androgen biodegradation pathway is functional in polluted ecosystems. Moreover, the distribution and abundance of androgen-degrading bacteria in the environment are yet to be investigated. In the present study, we examined microbial androgen degradation in the aerobic sewage of the Dihua Sewage Treatment Plant (DHSTP), which treats domestic wastewater produced by the three million residents of Taipei City, Taiwan. We used the following approaches: (*i*) identification of androgen metabolites through ultra-performance liquid chromatography - tandem mass spectrometry (UPLC-MS/MS), (*ii*) phylogenetic identification of the androgen degraders through Illumina Miseq sequencing, and (*iii*) detection of the essential catabolic gene *tesB* through PCR.

## Results

### UPLC-MS/MS identification of androgenic metabolites in DHSTP sewage

**Figure 2:**
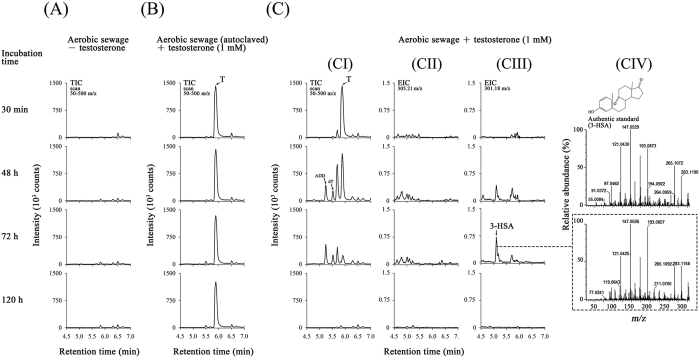
UPLC-APCI-MS/MS analysis of the ethyl acetate extracts of the DHSTP sewage treatment samples. (**A**) Total ion chromatograms of the active sewage incubated without testosterone. (**B**) Total ion chromatograms of the autoclaved sewage incubated with testosterone. (**C**) The active sewage incubated with testosterone. (CI) Total ion chromatograms of the androgen metabolites. (CII) Extracted ion chromatograms for 2, 3-SAOA (*m*/*z *= 305.21; expected retention time at 4.87 min) in the testosterone-treated sewage. (CIII) Extracted ion chromatograms for 3-HSA (*m*/*z *= 301.18) in the testosterone-treated sewage. (CIV) The MS/MS fragmentation spectra of the authentic standard (top) and the 3-HSA extracted from the testosterone-treated sewage (bottom). Abbreviations: ADD, androsta-1, 4-diene-3, 17-dione; dT, 1-dehydrotestosterone; 3-HSA, 3-hydroxy-9, 10-*seco*-androsta-1, 3, 5(10)-triene-9, 17-dione; 2, 3-SAOA, 17-hydroxy-1-oxo-2, 3-*seco*-androstan-3-oic acid; T, testosterone.

Androgenic metabolites were extracted from various sewage treatment samples and identified through UPLC-atmospheric pressure chemical ionization (APCI)-MS/MS (Fig. 2). No testosterone was detected in the original DHSTP sewage (Fig. 2A). Furthermore, testosterone was not degraded when incubated with autoclaved sewage (Fig. 2B). By contrast, testosterone was transformed to 1-dehydrotestosterone, AD, and ADD during the first two days of aerobic incubation of active sewage (Fig. 2CI). After 72 hours of incubation, the intensities of peaks corresponding to the androgens decreased considerably. We then used the extracted ion current at *m/z* 301.18 (the predominant ion peak of 3-HSA) to detect 3-HSA, the signature metabolite of the 9, 10-*seco* pathway, in the aerobic sewage (Fig. 2CIII). The UPLC retention time (5.10 min; Fig. 2CIII) and MS/MS fragmentation spectrum (Fig. 2CIV) of the extracted ion was comparable with that of the authentic standard.

An alternative pathway for androgen catabolism, the steroid 2, 3-*seco* pathway, was identified in some denitrifying bacteria^16^,^38^. Among them, at least *Sterolibacterium denitrificans* was reported to aerobically degrade testosterone through the 2, 3-*seco* pathway^37^. The ring-cleaved intermediate, 17-hydroxy-1-oxo-2, 3-*seco*-androstan-3-oic acid (2, 3-SAOA), was detected in the testosterone-treated denitrifying sewage^16^. We used the extracted ion current at *m*/*z* 305.21 (the predominant ion peak of 2,3-SAOA) to detect this compound, and no corresponding peak was present in the aerobic sewage incubated with testosterone (Fig. 2CII). The sewage treatments were performed in duplicate. 3-HSA, but not 2,3-SAOA, was detected in both replicates (Fig. 2 and S1).

**Figure 3:**
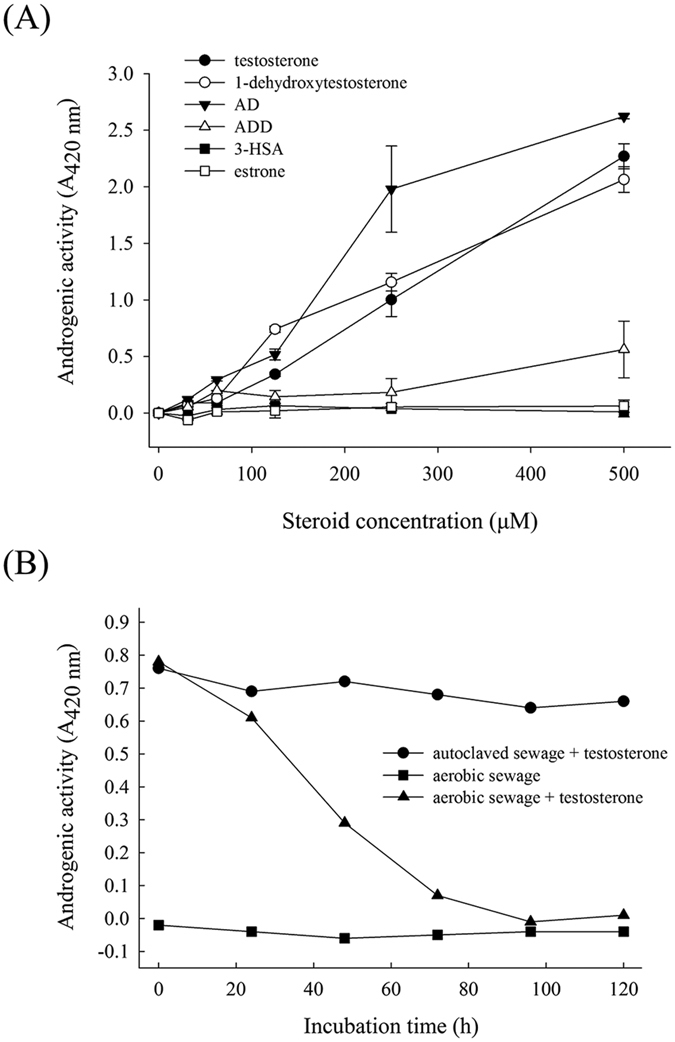
(**A**) The yeast androgen bioassay results of the individual intermediates of the 9, 10-*seco* pathway. The results are from one representative of three individual experiments. (**B**) The time course of androgenic activities in the negative control (testosterone-treated autoclaved sewage) and two treatments of the aerobic DHSTP sewage. The *A*_420_ of the solvent, DMSO (1% v/v), was set to zero. Data are shown as the mean ± SE of three experimental measurements.

We determined the androgenic activity of the initial intermediates of the 9, 10-*seco* pathway by using a *lacZ*-based yeast androgen assay. The results showed that testosterone, 1-dehydrotestosterone, AD, and ADD exhibited apparent androgenic activity. However, the secosteroid 3-HSA had no detectable androgenic activity even at a concentration of 500 μM (Fig. 3A). The androgenic activity of the ethyl acetate extracts from the sewage treatment samples was then determined (Fig. 3B). The androgenic activity of the sewage extracts decreased over time, which is consistent with the results of the androgen metabolite analysis.

### Phylogenetic identification of androgen-degrading bacteria in aerobic sewage

**Figure 4:**
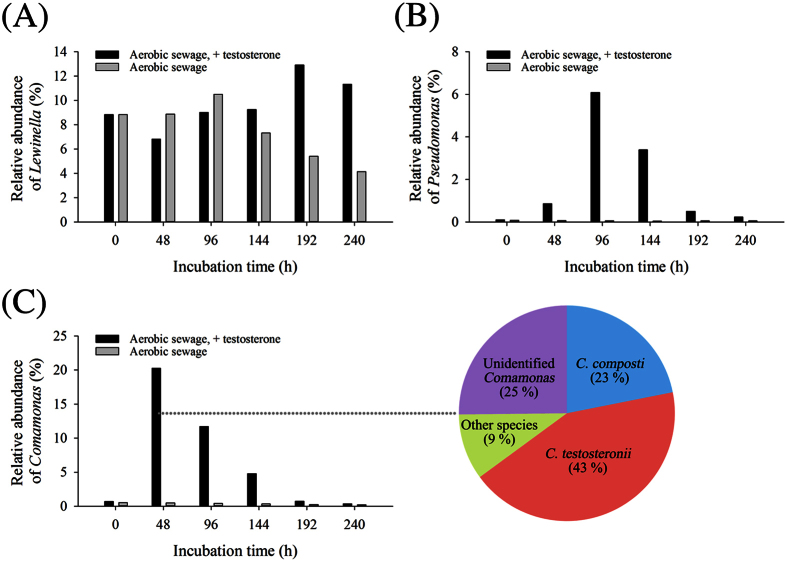
Genus-level phylogenetic analysis (Illumina MiSeq) revealed the temporal changes in the bacterial community structures in various aerobic sewage treatment samples. (**A**) *Lewinella* was commonly detected in all sewage treatments. (**B**) *Pseudomonas* was slightly enriched in the testosterone-treated aerobic sewage. (**C**) *Comamonas* was highly enriched in the testosterone-treated aerobic sewage. The pie chart represents the relative abundances of individual *Comamonas* spp. (100%) in the sewage incubated with testosterone for 48 hours. See Supplementary Fig. S2 for detailed information.

DNA was extracted from various sewage treatment samples. The V3-V4 hypervariable region of bacterial 16S rRNA gene was amplified through PCR, and the resulting amplicons were sequenced using an Illumina MiSeq sequencer (Illumina; San Diego, CA, USA). The sequences were analyzed using BaseSpace 16S Metagenomics App V1.01 (Illumina; San Diego, CA, USA) (Fig. 4). The nucleotide sequence data set was deposited in the National Center for Biotechnology Information (NCBI) Sequence Read Archive (accession number: SRP062202). From each sample, an average of 306 025 reads was obtained. The duplicates of individual sewage treatment samples exhibited high similarity in bacterial community structure (Fig. S2). Except the unclassified and other (individual genus with a relative percentage of <1%) microorganisms, 35 genera were identified in the DHSTP sewage, among which *Lewinella*, with a relative abundance between 4% and 13%, was present in all sewage treatment samples, regardless of the incubation conditions (Fig. 4A). Moreover, we observed no apparent enrichment of *Lewinella* spp. in the testosterone-incubated sewage.

Members of the genus *Pseudomonas* were slightly enriched in the testosterone-treated aerobic sewage (Fig. 4B). In a replicate, the relative abundance of *Pseudomonas* spp. reached 6% after 96 hours of incubation. The bacterial community analysis suggested that *Pseudomonas entomophila* and *P. panipatensis* were the most enriched species (Fig. S3). *Pseudomonas* spp. were not enriched in aerobic sewage incubated without testosterone (Fig. 4B), suggesting that *Pseudomonas* spp. might play a role in aerobic androgen biodegradation.

*Comamonas* spp. were enriched in the testosterone-treated aerobic sewage in the first two days, and their relative abundance accounted for approximately 20% of the total bacterial community (Fig. 4C). Further phylogenetic analysis suggested that after 48 hours of incubation with testosterone, most of the obtained sequences were associated with *C. testosteroni* (43% in the genus of *Comamonas*) and *C. composti* (23%) (Fig. 4C). The relative abundance of *Comamonas* spp. in the bacterial community then decreased with time and they were undetectable after 240 hours of incubation. *Comamonas* spp. were not enriched in the aerobic sewage incubated without testosterone (Fig. 4C). These results suggest that *Comamonas* spp. are active catabolic players in the testosterone-treated sewage. The enrichment of *Comamonas* spp. and *Pseudomonas* spp. were observed in the experimental replicates (Fig. S4). After 48 hours of aerobic incubation, the relative abundance of *Comamonas* spp. and *Pseudomonas* spp. reached 29% and 2%, respectively in the testosterone-treated sewage.

### PCR amplification of tesB-like genes in aerobic sewage

The catabolic gene (*tesB*) encoding the 3, 4-dihydroxy-9, 10-*seco*-androsta-1, 3, 5(10)-triene-9, 17-dione (3, 4-DHSA) dioxygenase of *C. testosteroni* TA441 was used as a query to blast UniProtKB/TrEMBL, and a selection of the BLASTp hits from the database is shown in Table 1. The most similar sequences belonged to betaproteobacteria and gammaproteobacteria. In known steroid-degrading proteobacteria, *tesB*-like genes are harbored in the conserved testosterone-degradation gene cluster^25^. Phylogenetic analysis (Fig. S5) revealed that the 3, 4-DHSA dioxygenases of proteobacteria tend to cluster and apparently differ from those of the actinobacterial genera *Gordonia*, *Mycobacterium*, *Nocardia*, and *Rhodococcus*, which can degrade steroids through the 9, 10-*seco* pathway^37^,^40^,^41^.

**Figure 5:**
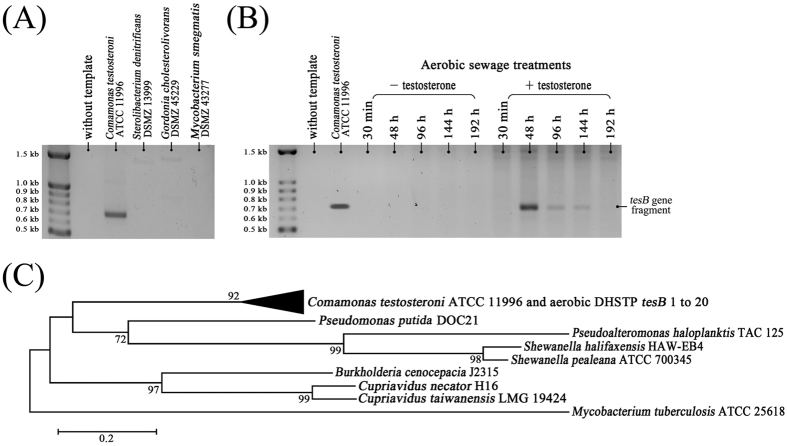
PCR-based functional assay with degenerate primers (see Fig. 7C for sequences) derived from the proteobacterial tesB genes. (**A**) Agarose gel electrophoresis revealed that a proteobacteria-specific *tesB* gene probe can be used to amplify the corresponding genes of androgen-degrading aerobes such as *C. testosteroni*. (**B**) *tesB*-like PCR products increased only in the testosterone-treated aerobic sewage. The agarose gel images shown in Fig. 5A,B were cropped to show the relevant data only. The full-length gels are present in Fig. S10. (**C**) The phylogenetic tree of the *tesB* genes obtained from aerobic sewage incubated with testosterone for 48 hours. Refer to Fig. S6 for the *tesB* sequences amplified from the aerobic sewage. The sequence of a gene encoding 3,4-DHSA dioxygenase from *M. tuberculosis* ATCC 25618 served as an outgroup sequence.

The specificity of degenerate primers was determined using genomic DNA isolated from several bacteria as templates. PCR products (approximately 700 bp) were amplified from the strictly aerobic *C. testosteroni* but not from the denitrifying *Sterolibacterium denitrificans* (Fig. 5A). No PCR products could be amplified from steroid-degrading actinobacteria (Fig. 5A). This could be because of the low sequence similarity of 3, 4-DHSA dioxygenases between actinobacteria and proteobacteria (Fig. S5).

When DNA isolated from the testosterone-treated sewage was used as a template, the time course changes in the amount of *tesB* sequences (Fig. 5B) corresponded with the temporal changes in *Comamonas* abundance in the aerobic sewage (Fig. 4C). No PCR products could be amplified from DNA isolated from sewage incubated without testosterone. PCR products amplified from the aerobic sewage (48 hours of incubation with testosterone) were cloned in *Escherichia coli*, and 20 clones were randomly selected for sequencing. All the obtained DNA fragments (Fig. S6, nucleotide sequences) exhibited the highest similarities (87–96%) to the *tesB* gene of *C. testosteroni* (Fig. 5C). The amplified *tesB* sequences obtained from another testosterone-treated sewage replicate were also highly similar to that of *C. testosteroni* (Fig. S7; see Fig. S8 for individual *tesB* sequences).

### Quantitative PCR confirmed the remarkable increase of the 16S rRNA and catabolic genes of C. testosteroni in the testosterone-treated sewage

**Figure 6:**
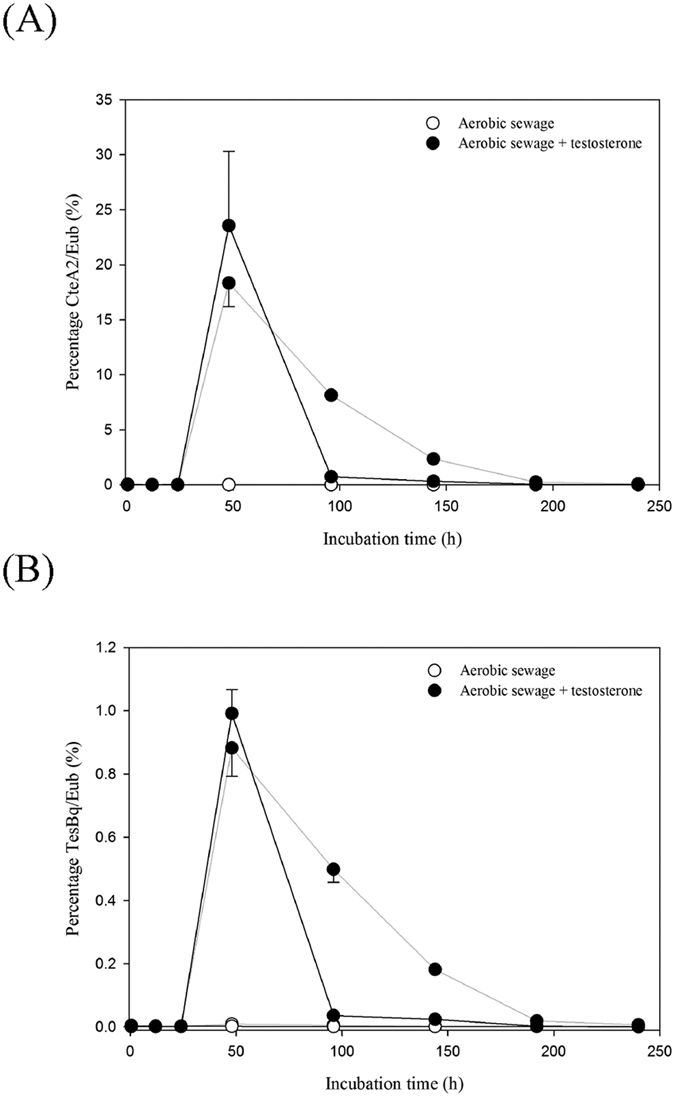
Real-time quantitative PCR indicated the temporal changes in the *C. testosteroni* 16S rRNA (**A**) and *tesB* (**B**) gene copies in the testosterone-treated sewage. Relative abundance of individual *C. testosteroni* genes was calculated as a proportion of the total number of bacterial 16S rRNA gene copies. Sewage treatments were performed in duplicate and the grey (Replicate 1) and black (Replicate 2) lines represent different replicates. Data are shown as the mean ± SE of three experimental measurements. Primer pairs CteA2, TesBq, and Eub were used to amplify the 16S rRNA and *tesB* genes of *C. testosteroni* as well as total eubacterial population, respectively. Real-time quantitative PCR standard curves obtained using the three primer pairs are shown in Fig. S11.

The metagenomic analysis (Fig. 4) and PCR-based functional assay (Fig. 5) could not robustly support an increase of the *C. testosteroni* population in the testosterone-treated sewage. Therefore, we performed a quantitative PCR study to examine the temporal changes in the 16S rRNA and *tesB* genes of *C. testosteroni* in different sewage treatments. The abundance of the *C. testosteroni* genes in each sample was normalized by the total eubacterial 16S rRNA gene. The duplicates of individual sewage treatment samples exhibited high similarity, and the apparent increase of the *C. testosteroni* genes was only observed in the testosterone-treated sewage (Fig. 6). The real-time quantitative PCR results were coherent with those of the conventional PCR assays. The relative abundance of the *C. testosteroni* 16S rRNA and *tesB* genes increased after 48 hours of incubation and decreased thereafter. After 48 hours of aerobic incubation, the abundances of the 16S rRNA (Fig. 6A) and *tesB* genes (Fig. 6B) in the duplicates reached 18.3~23.5% and 0.9~1.0%, respectively.

## Discussion

Activated sludge processes are used to treat wastewater in most cities in developed countries. The basic activated sludge process involves using a microbial community to mineralize organic carbons and oxidize ammonia (through nitrification) under aerobic conditions. Microbial communities play a crucial role in bioprocesses such as wastewater treatment and soil remediation^42^,^43^. However, exploiting the microbial resources requires an understanding of not only their phylogeny, but also their metabolic functions and ecological roles. Steroid hormones have been recognized as a major group of endocrine-disrupting chemicals, and sewage treatment plants play a critical role in removing these highly bioactive compounds^9^,^16^. In the present study, to explore the underlying biochemical mechanisms and microorganisms involved in androgen degradation in aerobic sewage, we applied various isotope-independent approaches, including the UPLC-MS/MS-based detection of the signature metabolites, community structure analysis, and PCR-based functional assays.

The 9, 10-*seco* pathway has been demonstrated in proteobacteria^25^ and actinobacteria^27^,^36^. We assigned 3-HSA as the signature metabolite of this aerobic degradation pathway because (*i*) 3-HSA does not possess a sterane structure and exhibits extremely weak androgen activity. Thus, 3-HSA detection demonstrates androgen biodegradation in the studied ecosystems. (*ii*) Compared with other secosteroids in this aerobic pathway, 3-HSA often accumulates in androgen-degrading bacterial cultures^33^,^38^,^44^ and could be detected through UPLC-MS/MS. Unlike sterane-containing androgens (e.g., testosterone, 1-dehydrotestosterone, AD, and ADD), the androgen metabolite 3-HSA cannot be easily ionized through APCI. Therefore, we used an extracted ion current method to detect this secosteroid. In the testosterone-treated aerobic sewage, the androgen activity decreased remarkably with time. However, the androgen activity did not decrease in the autoclaved sewage, indicating that testosterone degradation in sewage is exclusively due to microbial activity. We detected the common androgen metabolites, including 1-dehydrotestosterone, AD, and ADD, during the first two days. The biotransformation of testosterone to these androgens has been widely reported in natural and engineered ecosystems such as soils^17^,^19^, wastewater treatment plants^18^, and river sediments^20^. Furthermore, the corresponding redox enzymes have been identified in microorganisms including bacteria^45^, yeast, and fungi^46^. Although the detected androgen metabolites, namely 1-dehydrotestosterone, AD, and ADD, may be produced mainly by bacteria, our data do not exclude the role of eukaryotic microorganisms in the redox biotransformation reactions.

The UPLC retention time and the MS/MS fragmentation spectrum of the identified metabolite are comparable to that of the authentic standard, suggesting the production of 3-HSA in the aerobic sewage incubated with testosterone for three days. 3-HSA, the key intermediate of the 9, 10-*seco* pathway^25^,^47^, has rarely been detected in environmental samples. Yang *et al*.^24^ reported the detection of this secosteroid in bacterial cultures enriched from swine manure. An alternative steroid catabolic pathway, 2, 3-*seco* pathway, is adopted by aerobically grown *Sterolibacterium denitrificans*^37^, and 1-dehydrotestosterone, AD, ADD also serve as the initial metabolites in this alternative pathway. However, we did not detect 2, 3-SAOA, the representative androgen metabolite, in the aerobic sewage treatment samples. The results indicate that the 9, 10-*seco* pathway is functional in aerobic androgen biodegradation in sewage.

The 9, 10-*seco* pathway was described only in bacteria^25^,^36^. Thus, we studied the changes in the bacterial community in various aerobic sewage treatment samples. In testosterone-treated sewage, the appearance of 3-HSA and decrease in androgenic activity were accompanied by the enrichment of proteobacteria in the bacterial community. Although the 3, 4-DHSA dioxygenase-dependent 9, 10-*seco* pathway has been commonly identified in various bacteria, including proteobacteria^25^ and actinobacteria^37^,^48^, our Illumina MiSeq data suggest that proteobacteria, including *Comamonas* and *Pseudomonas*, are the androgen degraders in aerobic DHSTP sewage. None of the actinobacterial genera showed a relative abundance of >1% in the initial aerobic sewage samples collected from the DHSTP. Moreover, we observed no enrichment of actinobacteria in the testosterone-treated sewage. A recent investigation that analyzed the microbial communities in 13 Danish wastewater treatment plants also revealed betaproteobacteria as the predominant components, whereas members of actinobacteria exhibited extremely low relative abundance^49^. Bacteria belonging to Saprospiraceae (mainly *Lewinella* spp.) were predominantly present in the aerobic sewage of DHSTP; however, their abundance did not apparently increase in the testosterone-treated sewage. To our knowledge, *Lewinella* spp. have not been identified as steroid degraders. Nevertheless, our current data cannot exclude that *Lewinella* spp. and actinobacterial species play a role, directly or indirectly, in androgen degradation in aerobic sewage. This is because (*i*) androgen catabolism does not necessarily result in an enrichment of the populations of the degraders, especially in a short-term incubation; and (*ii*) in aerobic sewage, an increase of the degrader population could be counteracted by removal processes like predation and viral lysis.

The Illumina Miseq analysis of 16S rRNA genes enriched in the testosterone-treated sewage suggested that *Pseudomonas* spp. (likely *P. entomophila* and *P. panipatensis*) play a role in aerobic androgen degradation. These two *Pseudomonas* species were not described as testosterone-utilizing bacteria. Moreover, most steroid catabolic genes, including the *tesB* gene, were not found in the genomes of these two *Pseudomonas* species^36^,^50^. Our *tesB* gene probe was derived from several steroid-degrading proteobacteria including *P. putida*. However, the 40 sequenced *tesB* fragments did not exhibit high similarity to that of any *Pseudomonas* species. It is worth noting that the *tesB* sequences were amplified from the sewage incubated for two days, in which the bacterial community was dominated by *Comamonas* spp., but not *Pseudomonas* spp. Accordingly, the enrichment of *Pseudomonas* spp. might be due to the indirect involvement in the bioprocess (e.g., feeding on metabolites excreted by the androgen degraders). Nevertheless, further investigation is necessary to elucidate the androgen degradation potential of *Pseudomonas* species enriched in the aerobic sewage.

The Illumina Miseq analysis revealed an apparent dominance of *Comamonas* spp. (likely *C. testosteroni* and *C. composti*) in the testosterone-treated sewage. This is in line with the observed increase in the relative abundance the *C. testosteroni tesB* gene after 48 hours of incubation with testosterone. However, the result of quantitative PCR revealed a much lower relative abundance for the *tesB* gene as compared to the *C. testosteroni* 16S rRNA gene. The lower abundance of the *C. testosteroni tesB* gene in the testosterone-treated sewage may be due to (*i*) multiple copies of the 16S rRNA gene in the bacterial chromosome, and (*ii*) the higher sequence diversity of the *tesB*-like sequences (87~96%), compared with that of the 16S rRNA gene (>98%).

*Comamonas* spp. were also enriched in testosterone-amended swine manure^24^. Thus, *Comamonas* species might play an important role in aerobic androgen degradation in the environment, such as in sewage and agricultural soil treated with manure. Members of the *Comamonas* genus belong to betaproteobacteria, with versatile metabolic capacities and possess a wide spectrum of substrate utilization. To date, the genus *Comamonas* encompasses 11 species that have been validated: *C. aquatica*, *C. badia*, *C. composti*, *C. denitrificans*, *C. kerstersii*, *C. koreensis*, *C. nitrativorans*, *C. odontotermitis*, *C. terrigena*, *C. testosteroni*, and *C. thiooxidans*^51^,^52^. These species exhibit extremely different physiological and metabolic capabilities. For instance, *C. thiooxidans* can grow under anoxic conditions, whereas other species are strictly aerobes^51^. Among them, only *C. testosteroni* was reported to utilize steroids as the sole carbon source^53^. The comparative genomic analysis also indicated that steroid catabolic genes are only present in the *C. testosteroni* strains, but not in other *Comamonas* species^36^. In the present study, although *C. composti* was assigned as an enriched species during aerobic incubation with testosterone, our metabolite analysis indicated that this species cannot degrade androgens (Fig. S9). Moreover, we did not find a *tesB*-like gene in the draft genome of *C. composti* (accession: NZ_AUCQ00000000). Liu *et al*.^52^ compared the genomes of 14 *C. testosteroni* strains, and steroid catabolic genes were found in all genomes. Considering that androgens are typically present at low concentrations (ng L^−1^–μg L^−1^) in the natural environment, it is unreasonable that *C. testosteroni* strains have evolved the unusual metabolic capability to use rare and structurally complex carbon sources such as testosterone. This bacterium can also grow on bile acids^32^, which often occur in significant amounts in the environment. One may thus envisage that bile acids could serve as the target substrates of the steroid catabolic enzymes of *C. testosteroni*.

*C. testosteroni* is the most widely studied microorganism for aerobic androgen degradation^25^. Here, for the first time, we provide strong evidence showing that *C. testosteroni* plays a role in removing androgens from the environment. Considering that the degradation of steroid hormones in anaerobic environments is typically slow^54^, aeration and introducing aerobic degraders could be efficient bioremediation options. Although *C. testosteroni* is not a dominant species (0.4% relative abundance in the initial sewage bacterial community) in the DHSTP sewage, which typically contains androgen concentrations of approximately 35 nM^16^, these bacteria can efficiently respond to changes in androgen input (1 mM in this study), suggesting that *C. testosteroni* could be used in the bioremediation of steroid-contaminated ecosystems.

The high-throughput sequencing of 16S rRNA gene has enabled a deeper understanding of bacterial diversity in complex environmental samples; however, the method also introduces ambiguity because of the limited taxonomic capability of short reads (450 bp in the present study). Moreover, the taxonomic assignments are inconsistent among different classification methods^55^. We identified several *Comamonas* species in the testosterone-treated sewage. However, *C. composti* showed no androgen-degrading ability. A recent genomic study also indicated that the distribution of the steroid degradation pathways among proteobacterial taxa is generally patchy, and only a few genomes from each proteobacterial genus appear to encode steroid catabolic genes^36^. Thus, our results suggest that the taxonomic assignment of bacteria based the high-throughput sequencing of 16S rRNA genes alone is insufficient for characterizing biodegradation events. The combination of 16S rDNA-based phylogenetic analysis, signature metabolite probing, and PCR-based functional detection proposed in this study may provide information on both the biochemical mechanisms and the active players in bacterial degradation. Future studies should include a kinetic analysis of substrate utilization by *C. testosteroni* and the immobilization of bacterial cells to improve the efficiency of androgen removal in sewage treatment plants. Moreover, systems biology approaches, such as metatranscriptomics or metaproteomics coupled with metagenomics, can elucidate the ecophysiological relevance of androgen-degrading microbes. This should also facilitate developing or engineering microbial consortia for the efficient removal of steroids from polluted ecosystems.

## Conclusions

The application of an integrated approach comprising several culture-independent tools appears useful for investigating the microbiology and biochemistry of environmentally relevant processes such as steroid biodegradation. Under our experimental conditions, aerobic androgen biodegradation in the testosterone-treated sewage proceeds through the established 9, 10-*seco* pathway. However, UPLC-MS/MS analyses cannot help to identify the metabolites from undescribed degradation pathways. Our data thus do not exclude the operation of other degradation pathways in the testosterone-treated aerobic sewage. The metegenomic analysis, PCR-based functional assay, and quantitative PCR supported the catabolic role of *C. testosteroni* in the testosterone-treated sewage. However, our data did not indicate the crucial role of *C. testosteroni* in aerobic androgen degradation in the operating sewage treatment plants where androgens are present at much lower concentrations.

## Materials and Methods

### Chemicals and bacterial strains

The chemicals were of analytical grade and were purchased from Fluka, Mallinckrodt Baker, Merck, and Sigma-Aldrich. *C. testosteroni* ATCC 11996 was obtained from the American Type Culture Collection (Manassas, VA, USA). *Gordonia cholesterolivorans* DSMZ 45229, *Mycobacterium smegmatis* DSMZ 43277, and *S. denitrificans* DSMZ 13999 were purchased from the Deutsche Sammlung für Mikroorganismen und Zellkulturen (Braunschweig, Germany). *S. denitrificans* was anaerobically grown on testosterone^56^. The other aerobic bacteria were aerobically cultured in Luria-Bertani medium.

### Collection of sewage samples

The DHSTP is the largest municipal wastewater treatment plant (500 000 m^3^ day^−1^) in Taipei. Along with domestic water, the DHSTP receives industrial, medical, and livestock wastewater as well as groundwater^57^. The design of the DHSTP includes an anoxic/oxic process for removing carbon and nitrogen, and the hydraulic retention time is approximately 10 hours^57^,^58^. Sewage samples (10 L) were collected from the aerobic tank of the DHSTP in June 2014. The aerobic sewage was placed in a sterilized 20-L glass bottle, and delivered to the laboratory within 30 minutes.

### Incubation of aerobic sewage with testosterone

The DHSTP aerobic sewage samples (0.5 L sewage in 2-L glass bottles) were incubated under the following conditions: autoclaved sewage with testosterone (1 mM), active sewage without testosterone, and active sewage with testosterone. The sewage treatments were performed in duplicate, and the bottles were incubated at 25 °C with stirring at 160 rpm for two weeks. Samples (10 mL) were withdrawn from the bottles every 12 hours and stored at −80 °C before use. The androgenic activity and androgen metabolites in the sewage samples were detected using the yeast androgen assay and UPLC-APCI-MS/MS, respectively. The bacterial 16S rRNA and functional *tesB* genes in the sewage samples were analyzed through Illumina MiSeq sequencing and PCR-based functional assays, respectively.

### UPLC-MS/MS identification of androgenic metabolites in sewage

Aerobic sewage samples (1 mL) were extracted three times with the same volume of ethyl acetate. The extracts were pooled, the solvent was evaporated, and the residues were re-dissolved in 100 μL of methanol. The ethyl acetate extractable samples were analyzed through UPLC-APCI-MS/MS, as described by Wang *et al*.^38^.

### lacZ-based yeast androgen bioassay

The sewage samples (0.5 mL) were extracted three times using equal volume of ethyl acetate. After the solvent evaporated, the extracts were re-dissolved in the same volume of dimethyl sulfoxide (DMSO), and the androgenic activity in the sewage samples was determined using a *lacZ*-based yeast androgen assay. The yeast androgen bioassay was conducted as described by Fox *et al*.^59^ with slight modifications. Briefly, the individual steroid standards or sewage extracts were dissolved in DMSO, and the final concentration of DMSO in the assays (200 μL) was 1% (v/v). The resulting DMSO solutions (2 μL) were added to yeast cultures (198 μL, initial OD_600nm_ = 0.5) located in a 96-well microtiter plate. The β-galactosidase activity was determined after 18 hours incubation at 30 °C. The yeast suspension (25 μL) was added to a Z buffer (225 μL) containing *o*-nitrophenol-β-D-galactopyranoside (2 mM), and the reaction mixtures were incubated at 37 °C for 30 min. The reactions were stopped by adding 100 μL of 1 M sodium carbonate, and the amount of yellow-colored nitrophenol product was determined spectrophotometrically at 420 nm on a plate spectrophotometer (SpectraMax M2e, Molecular Devices).

### Illumina MiSeq sequencing of bacterial 16S rRNA amplicons

DNA was extracted from the frozen sewage samples by using the Powersoil DNA isolation kit (MO BIO Laboratories). A 16S amplicon library was prepared according to the Illumina 16S Metagenomic Sequencing Library Preparation Guide (/mentation/chemistry_documentation/16s/16s-metagenomic-library-prep-guide-15044223-b.pdf) with slight modifications. Genomic sections flanking the V3-V4 region of the bacterial 16S rRNA gene were amplified from 24 sewage treatment samples by using HiFi HotStart ReadyMix (KAPA Biosystems) through PCR (95 °C for 3 minutes; 25 cycles: 95 °C for 30 s, 55 °C for 30 s, 72 °C for 30 s, and 72 °C for 5 min). A primer pair flanked by the Illumina Nextera linker sequence (forward primer: 5′-TCGTCGGCAGCGTCAGATGTGTATAAGAGACAGCCTACGGGNGGCWGCAG-3′ and reverse primer: 5′-GTCTCGTGGGCTCGGAGATGTGTATAAGAGACAGGACTACHVGGGTATCTAATCC-3′) was used. The PCR products were first separated on an agarose gel, and those with the expected size (approximately 445 bp) were excised from the gel and purified using the GenepHlow Gel/PCR kit (Geneaid). Next, Illumina Nextera XT Index (Illumina) sequencing adapters were integrated to the ends of the amplicons through PCR (95 °C for 3 min; 8 cycles: 95 °C for 30 s, 55 °C for 30 s, 72 °C for 30 s; and 72 °C for 5 min). The final libraries were purified using AMPure XP beads (Beckman Coulter) and quantified using the Qubit dsDNA HS Assay Kit (Life Technologies). The library profiles were randomly analyzed using the Agilent High Sensitivity DNA Kit on BioAnalyzer. To ensure consistency in pooling, all 24 libraries were subjected to quantitative PCR normalization by using KAPA Library Quantification Kits to derive the molar concentration, and the final library mixture was verified through quantitative PCR. The library pool was sequenced on the Illumina MiSeq V2 sequencer by using MiSeq Reagent Kit V3 for paired-end (2 × 300 bp). We analyzed the sequencing data in the Illumina BaseSpace cloud service by using the BaseSpace 16S Metagenomics App (Illumina) (http://support.illumina.com/content/dam/illumina-support/documents/documentation/software_documentation/basespace/16s-metagenomics-user-guide-15055860-a.pdf). The reads were classified against the Illumina-curated version of May 2013 Greengenes taxonomy database by using the Ribosomal Database Project (RDP) naïve Bayesian algorithm (http://rdp.cme.msu.edu/classifier/).

### PCR by using the tesB gene probe

**Figure 7:**
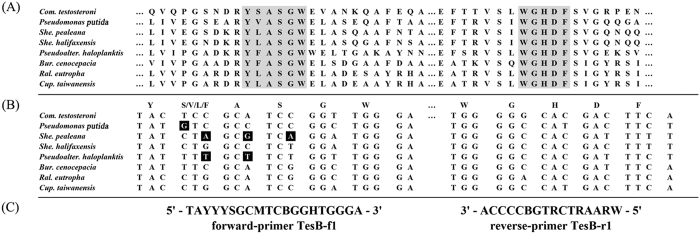
Multiple alignment of amino acid sequences of 3, 4-DHSA dioxygenase (TesB) showing conserved regions in the TesB proteins that were used to design a *tesB*-specific gene probe. (**A**) Comparison of the TesB sequences from androgen-degrading proteobacteria (Horinouchi *et al*.^25^): *Comamonas* (*Com*.) *testosteroni* TA 441, *Pseudomonas putida* DOC21, *Shewanella* (*She*.) *pealeana* ATCC 700345, *She*. *halifaxensis* HAW-EB4, *Pseudoalteromonas* (*Pseudoalter*.) *haloplanktis* TAC 125, *Burkholderia* (*Bur*.) *cenocepacia* J2315, *Ralstonia* (*Ral*.) *eutropha* H16, and *Cupriavidus* (*Cup*.) *taiwanensis* LMG 19424. (**B**) Conserved nucleotide sequence regions of the corresponding *tesB* genes. (**C**) Deduced primer pairs for detecting *tesB* genes. M = A + C, R = A + G, S = G + C, Y = C + T, W = A + T, B = T + G + C, and H = A + T + C. Reverse type (white on black) indicates mismatches to the degenerate primers.

Multiple alignment of the *tesB*-like genes from eight testosterone-degrading proteobacteria^25^ was performed using Align/Assemble (Genious 8.1.4). A degenerate primer pair [forward primer (*tesB*-f1): 5′–TAYYYSGCMTCBGGHTGGGA–3′ and reverse primer (*tesB*-r1): 5′–WRAARTCRTGBCCCCA–3′] were deduced from the conserved regions (Fig. 7). Furthermore, the *tesB* fragments were amplified through PCR (94 °C for 2 minutes; 30 cycles: 94 °C for 30 s, 48 °C for 30 s, 72 °C for 60 s; and 72 °C for 10 minutes). The partial *tesB* sequences (approximately 700 bp) amplified from the aerobic sewage were cloned in *E. coli* (One Shot TOP10; Invitrogen) by using the pGEM-T Easy Vector Systems (Promega). The *tesB* genes were sequenced on a ABI 3730xI DNA Analyzer (Applied Biosystems; Carlsbad, CA, USA) with BigDye terminator chemistry according to the manufacturer’s instructions.

### Real-time quantitative PCR

Specific primer pair CteA2^60^ [forward primer (CteA2-for): 5′–TTGACATGGCAGGAACTTACC–3′ and reverse primer (CteA2-rev): 5′–TCCCATTAGAGTGCTCAACTG–3′] and general primer pair Eub^61^ [forward primer (341F): 5′–CCTACGGGAGGCAGCAG–3′ and reverse primer (534R): 5′–ATTACCGCGGCTGCTGGC–3′] were used to amplify the 16S rRNA gene of *C. testosteroni* and total eubacterial population, respectively. The *tesB*-specific primer pair TesBq [forward primer (TesBq-f1): 5′–GCAAAAGAGCCAGGTCAAGCT–3′ and reverse primer (TesBq-r1): 5′–GCCGCCATAGCCGAACT–3′] was derived from the conserved regions the 40 *tesB* fragments (Figs S6 and S8). Three replicates of real-time quantitative PCR experiments were performed using an ABI 7300 Sequence Detection System (Applied Biosystems). The PCR mixture (20 μL) contained 10 μL of Power SYBR Green PCR master mix (Applied Biosystems), 0.1 μM for each primer, and 30 ng of environmental DNA. The thermal cycling conditions consisted of an initial denaturation step of 95 °C for 10 min, followed by 40 cycles of 95 °C for 15 s and 60 °C for 60 s.

## Additional Information

**How to cite this article**: Chen, Y.-L. *et al*. Identification of *Comamonas testosteroni* as an androgen degrader in sewage. *Sci. Rep.* **6**, 35386; doi: 10.1038/srep35386 (2016).

## Supplementary Material

Supplementary Information

## Figures and Tables

**Figure 1:**
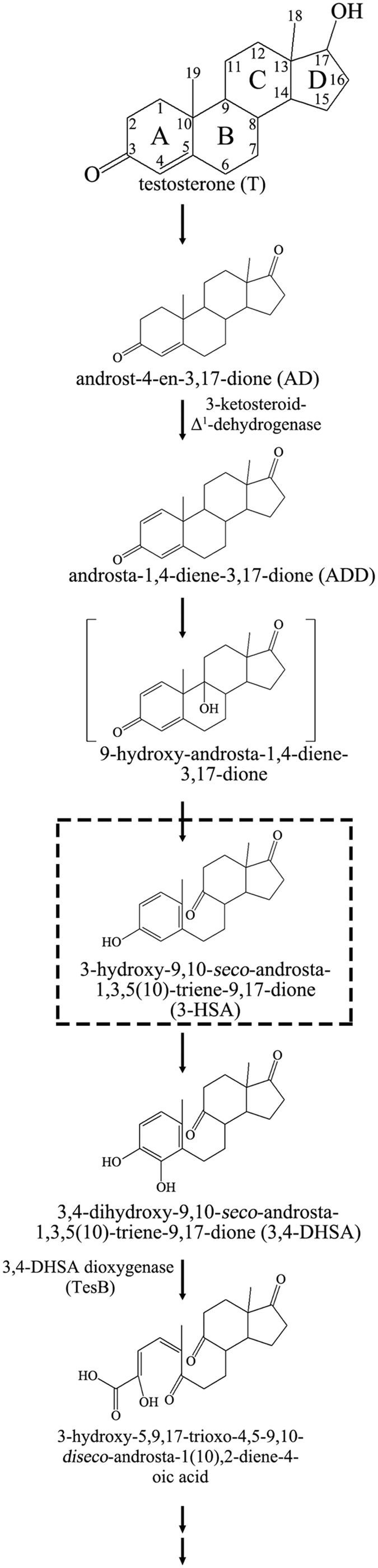
Simplified aerobic catabolic pathway of testosterone (9, 10-*seco* pathway) in *C. testosteroni* TA441. The compound in bracket is presumed. The suggested signature metabolite is enclosed in box.

**Table 1:** 3, 4-DHSA dioxygenases used for constructing the phylogenetic tree shown in Fig. S4.

Microorganism	Group	Sequence identity (%)	Accession
*Comamonas testosteroni* TA441	β-proteobacteria	100.0%	Q9FAE3
*Pseudomonas putida* DOC21	γ-proteobacteria	62.2%	H9ZGL6
*Shewanella pealeana* ATCC 700345	γ-proteobacteria	61.7%	A8H4I6
*Pseudoalteromonas haloplanktis* TAC 125	γ-proteobacteria	59.7%	Q3IE83
*Burkholderia cenocepacia* J2315	β-proteobacteria	59.1%	B4EKN7
*Ralstonia eutropha* (*Cupriavidus necator*) H16	β-proteobacteria	58.8%	F8GRC0
*Cupriavidus taiwanensis* LMG 19424	β-proteobacteria	58.4%	B2AIU1
*Rhodococcus jostii* RHA1	actinobacteria	44.5%	Q9KWQ5
*Gordonia polyisoprenivorans* DSM 44266	actinobacteria	44.1%	H6MSZ4
*Amycolicicoccus subflavus* DSM 45089	actinobacteria	44.1%	F6EN61
*Amycolatopsis decaplanina* DSM 44594	actinobacteria	44.0%	M2WZF0
*Amycolatopsis mediterranei* U-32	actinobacteria	43.8%	D8HWH1
*Kutzneria* sp. 744	actinobacteria	43.3%	W7SSZ6
*Rhodococcus pyridinivorans* AK37	actinobacteria	43.1%	H0JLI7
*Gordonia terrae* C-6	actinobacteria	42.5%	R7YBG9
*Dietzia cinnamea* P4	actinobacteria	42.2%	E6J6R1
*Mycobacterium tuberculosis* ATCC 25618	actinobacteria	42.1%	I6YCG0
*Nocardia seriolae*	actinobacteria	41.3%	A0A0B8MZT4
Pseudomonas pseudoalcaligenes*	γ-proteobacteria	39.7%	P08695

The sequences were retrieved from the UniProtKB/TrEMBL database by performing standard BLASTP search with 3, 4-DHSA dioxygenase (the product of *tesB*) from *C. testosteroni* TA441 as a query. In known steroid-degrading proteobacteria, the dioxygenase gene is harbored in the conserved testosterone-degradation gene cluster^25^.

^*^The biphenyl-2, 3-diol 1, 2-dixoxygenase of *P. pseudoalcaligenes.*
