# Supplementary material for: Identification of Comamonas testosteroni as an androgen degrader in sewage
Source: Sci Rep. 2016 Oct 13;6:35386. doi: 10.1038/srep35386 (PMC5062160; doi:10.1038/srep35386)
Supplement: Supplementary Information [file srep35386-s1.pdf]

Identification of *Comamonas testosteroni* as an androgen degrader in sewage

Running title: Aerobic androgen degradation in sewage

Yi-Lung Chen,[1,2,3†] Chia-Hsiang Wang,[1†] Fu-Chun Yang,[1] Wael Ismail,[4] Po-Hsiang Wang,[1#] Chao-Jen Shih,[1] Yu-Ching Wu,[5] and Yin-Ru Chiang[1,2,3*]

[1]Biodiversity Research Center, Academia Sinica, Taipei, 115, Taiwan.

[2]Department of Life Science, National Taiwan Normal University, Taipei, 106, Taiwan.

[3]Biodiversity Program, Taiwan International Graduate Program, Academia Sinica and National Taiwan Normal University, Taipei, 115, Taiwan.

[4]Environmental Biotechnology Program, Life Sciences Department, College of Graduate Studies, Arabian Gulf University, Manama, 26671, Kingdom of Bahrain.

[5]Institute of Plant and Microbial Biology, Academia Sinica, Taipei, 115, Taiwan.

[†]Y.-L. Chen and C.-H. Wang contributed equally to this study.

#Current address: Department of Chemical Engineering and Applied Chemistry, University of Toronto, Toronto, Ontario, Canada

*For correspondence. Y.-R. Chiang, Biodiversity Research Center, Academia Sinica, 128 Academia Road Sec. 2, Nankang, Taipei 115, Taiwan. E-mail: yinru915@gate.sinica.edu.tw; Tel. (+886) 2 2787 2251; Fax (+886) 2 2789 9624.

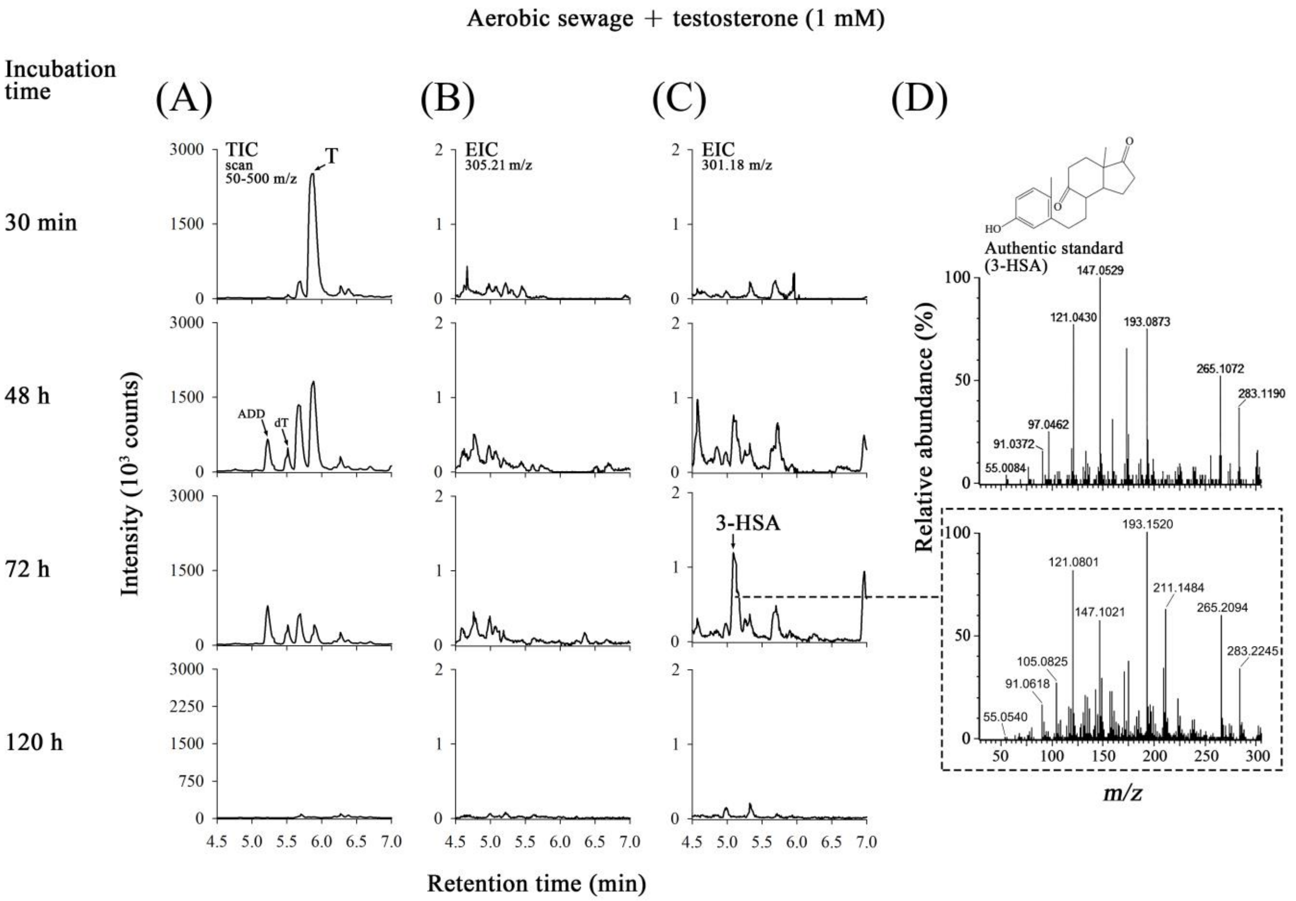

Figure S1. UPLC-APCI-MS/MS analyses of the ethyl acetate extracts of testosterone-amended sewage Replicate 2. (A) Total ion chromatograms (TIC) of the androgen metabolites. Extracted ion chromatograms for (B) 2,3-SAOA (*m/z* = 305.21) and for (C) 3-HSA (*m/z* = 301.18) in ethyl acetate extracts of the testosterone-incubated sewage. The expected UPLC retention time of 2,3-SAOA is 4.87 min. (D) The MS/MS spectra of the authentic standard (top) and 3-HSA extracted from the testosterone-incubated active sewage (bottom). Abbreviations: ADD, androsta-1,4-diene-3,17-dione; dT, 1-dehydrotestosterone; 3-HSA, 3-hydroxy-9,10-seco-androsta-1,3,5(10)-triene-9,17-dione; 2,3-SAOA, 17-hydroxy-1-oxo-2,3-*seco*-androstan-3-oic acid; T, testosterone.

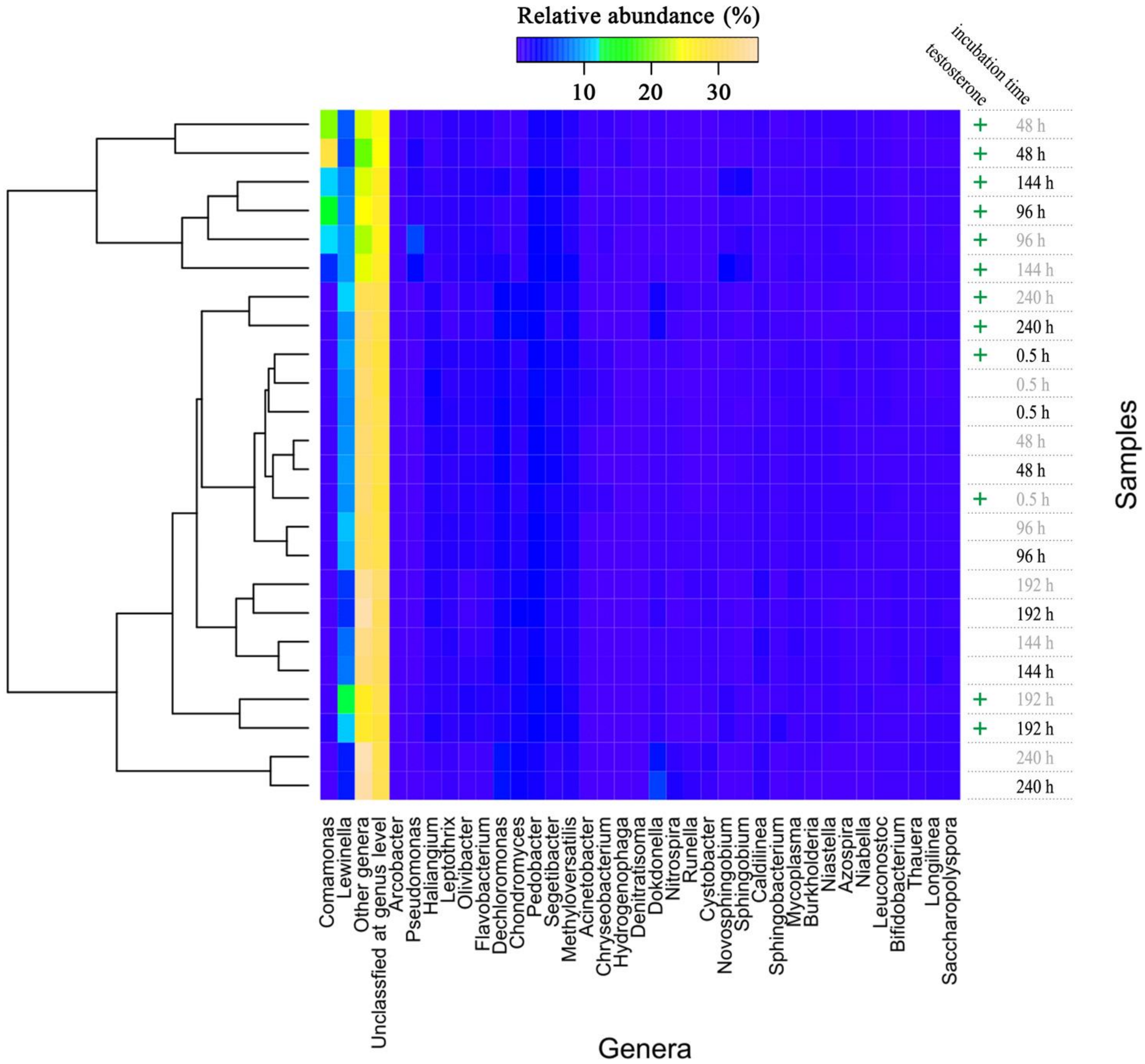

Figure S2. The heatmap represents the relative abundances of the indicated genera in the microbiota obtained from individual sewage treatment samples at a single time point. Sewage treatments were performed in duplicate and the individual incubation time labeled grey (Replicate 1) and black (Replicate 2) represents different replicates. Individual genera with a relative percentage of <1% are grouped as “other genera.” The dendrogram represents the average linkage hierarchical clustering of the sewage samples based on the Bray-Curtis dissimilarity matrix.

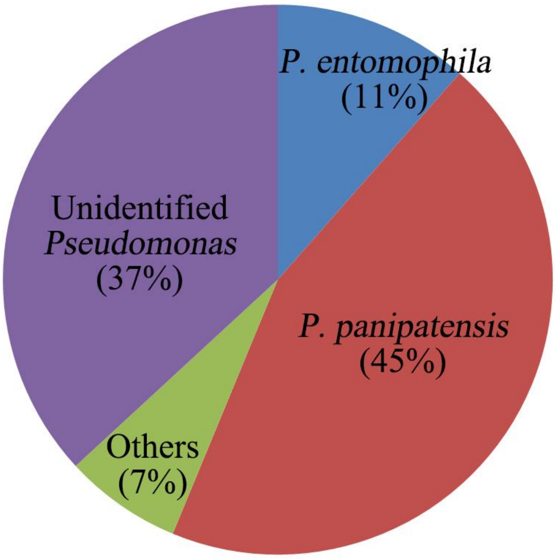

Figure S3. The pie chart represents the relative abundances of individual species in the genus of *Pseudomonas* (100%) in the aerobic DHSTP sewage incubated with testosterone (1 mM) for 96 hours.

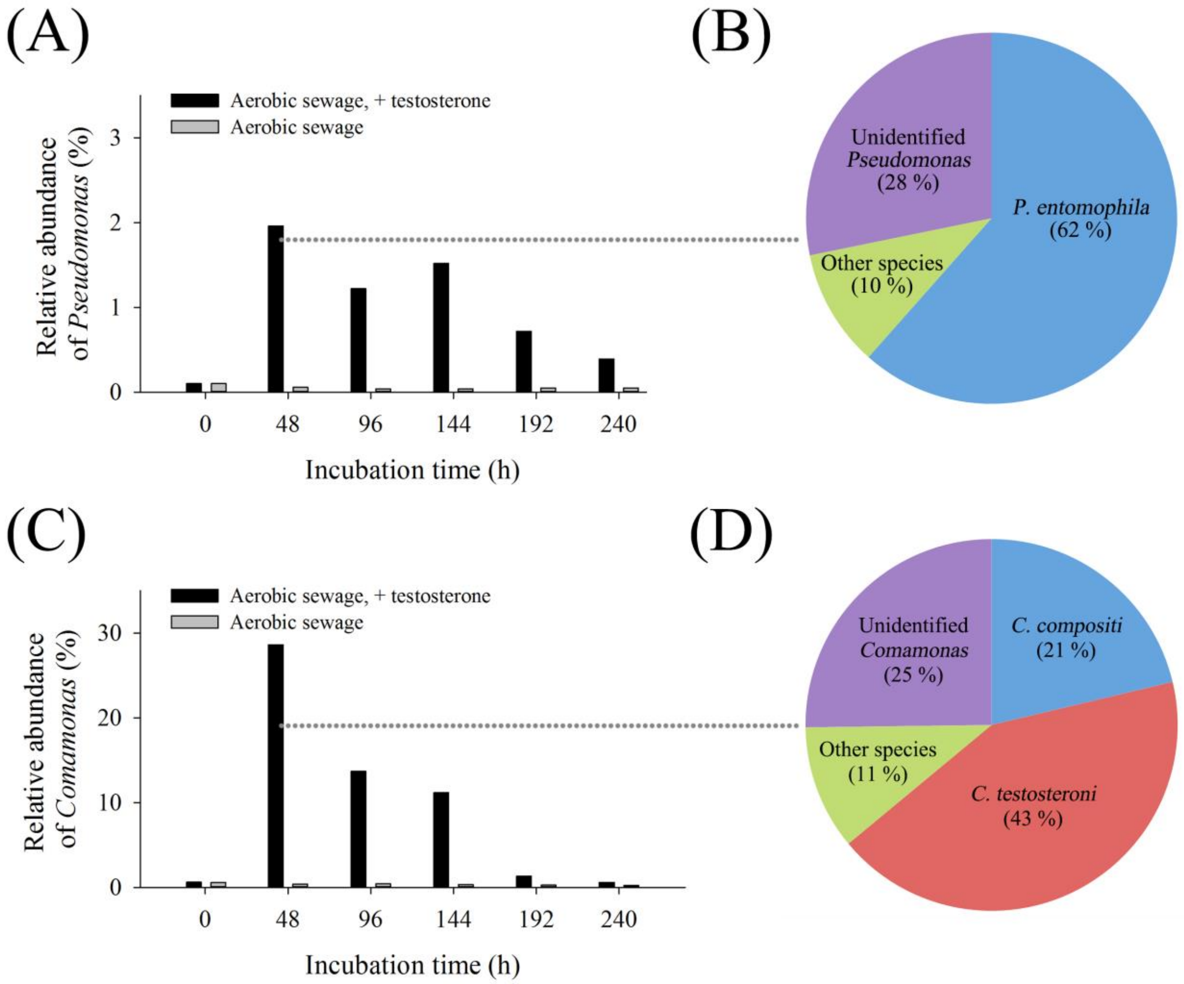

Figure S4. Temporal changes in the bacterial community structures in sewage Replicate 2. (A) Slight increase of of *Pseudomonas* spp. in the aerobic sewage incubated with testosterone (1 mM). (B) The pie chart represents the relative abundances of individual *Pseudomonas* spp. (100%) in the sewage incubated with testosterone for 48 hours. (C) Significant increase of *Comamonas* spp. in the aerobic sewage incubated with testosterone. (D) The pie chart represents the relative abundances of individual *Comamonas* spp. (100%) in the sewage incubated with testosterone for 48 hours.

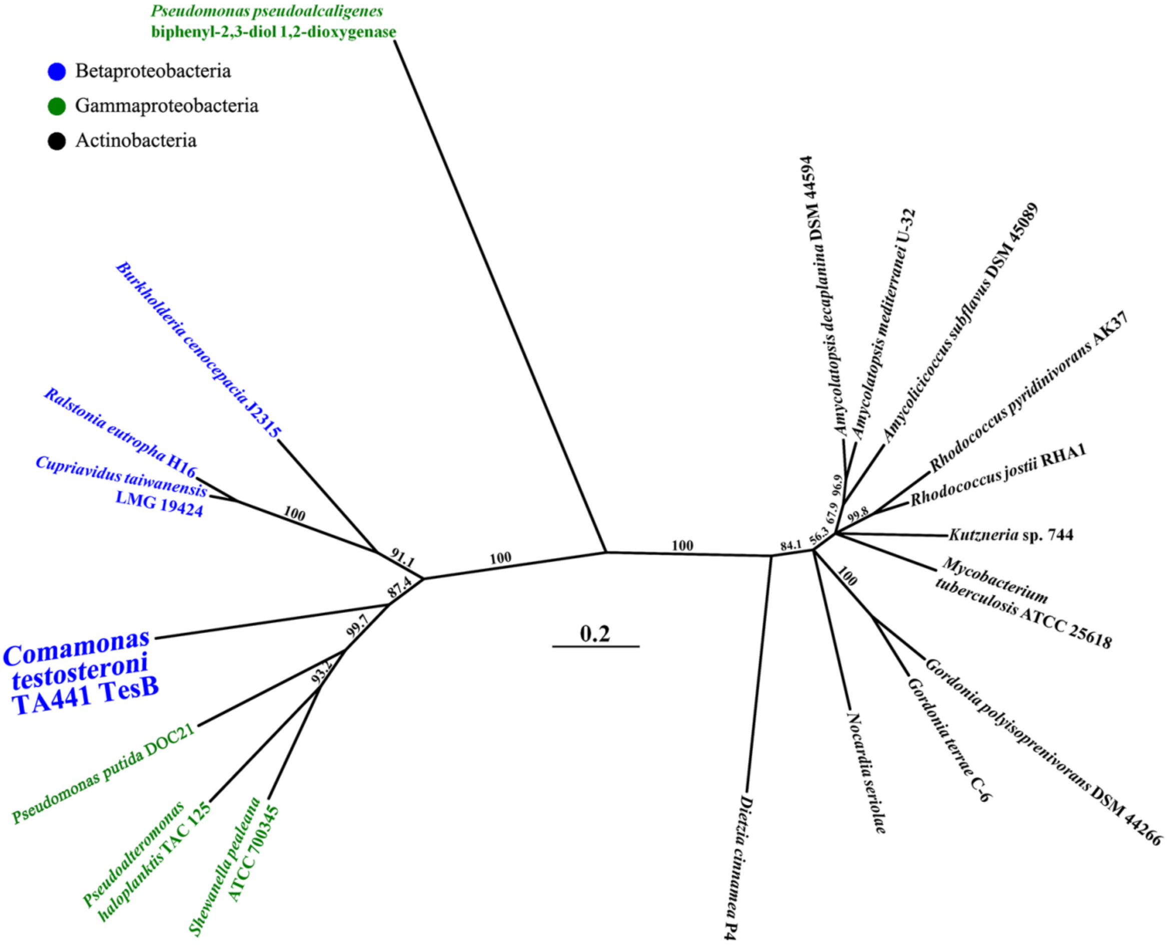

Figure S5. Phylogenetic tree of 3,4-DHSA dioxygenases involved in the *meta*-cleavage of the steroidal A-ring. The primary structure of biphenyl-2,3-diol 1,2-dioxygenase from *P. pseudoalcaligenes* served as an outgroup sequence. Phylogenetic trees were constructed using the neighbor-joining method with the Jukes-Cantor parameter and a bootstrap value of 1000.

Figure S6. Twenty *tesB* DNA fragments obtained from the aerobic DHSTP sewage (incubated with testosterone for 48 hours ; Replicate 1) by using *tesB*-specific degenerate primers *tesB*-f1/*tesB*-r1.

>Aerobic DHSTP *tesB*_1
AAGTGGCCAACAAGGAAGCTTTTGACAAAGCGCTGAAGGTACTGGAGGCGGCTGACGT
GCGCTATGAAATGGGCAGCGCCGAGCTGTGCACCAAGCGCCGCGTGCAGCAACTGGCG
GTGGTGATCGATCCTTCGGGCAACCGCCACGAAATCGTCTGGGGCTTCAAGTCCGATTT
CATCCACTTTGCCTCGCCGCAAGGCGTTTCCCGCTTCATCACGGGTGACTACGGCCTGG
GCCACACCGTGCTGCCCGCTCCCGACTTTGAAAAGACCGTGGCTTTTGTGCGCAATGTC
CTGGGTTTTGGTCTGTCGGACATCTACAACTTCAAGCCCGCTGGTGATGCAGGCCCCAC
GGTTCGCATTCACTTCTTCCACTGCGCCAATGGCCGCCACCACAGTCTGGCGCTGGCCG
AGTTCCCCTCGCCCACAGGCTGCGTGCACGTGATGGTGGAAGTGGACAACATGCCCGA
AGTGGGCCGTGCCATGGACCGCATGCAAAAGAGCCAGACCAAGCTGTCGGCCACGCTG
GGCCAGCACACCAACGACAAGATGATTTCCTTCTACATGAAGACGCCCTCGAACTTCGA
TCTGGAGTTTGGCTACGGCGGCGCAATCATCGACTGGGATCACCACATCACCCACGAAT
TCACGACCGTAAGTCTCTGGGGCCACGATTTT

>Aerobic DHSTP *tesB*_2
GGCATCTGGTTGGGAGGTGGCCAACAAGCAAGCCTTCGAGCAGGCGCTCGAGGTGTTG
CAGGCCGCTGATGTGCACTACGAAATGGGCCGCCCCGAGCTGTGTGCCAAGCGCCATGT
GCAGGAGTTGGCCATCGTGATGGATCCGTCCGGAAACCGTCATGAGATCGTCTGGGGCT
TCAAGTCCGACTTCAGCCATTTCGCATCGCCCCAGGGCGTGTCGCGCTTCATCACCGGT
GACTATGGTCTGGGCCACACCGTGCTGCCTGCTCCCGACTTTGACAAGACCGTGGCCTT
TGTGCGCAATGTGCTGGGCTTTGGCCTCTCGGATATTTACAACTTCAAGCCCGCAGGCGA
TGCCGGCCCCACGGTCCGAATCCACTTCTTCCACTGCGCCAACGCTCGTCACCACAGCC
TGGCGCTGGCCGAATTCCCTTCTCCCTCGGGTTGCGTGCACGTGATGGTGGAGGTGGAC
AACATGCCCGAAGTGGGCCGTGCCATGGACCGCATGCAAAAGAGCCAGGTCAAGCTCT
CTGCCACTCTGGGTCAGCACACCAACGACAAGATGATCTCGTTCTACATGAAGACGCCC
TCCAACTTCGACCTGGAGTTCGGCTATGGCGGCGCCATCATCGGCTGGGAACACCACAT
CACGCACGAGTTCACGACCGTAAGC

>Aerobic DHSTP *tesB*_3
AGGTGGCCAACAAGCAAGCCTTCGAGCAGGCGCTCGAGGTGTTGCAGGCCGCTGATGT
GCACTACGAAATGGGCCGCCCCGAGCTGTGTGCCAAGCGCCATGTGCAGGAGTTGGCC
ATCGTGATGGATCCGTCCGGAAACCGTCATGAGATCGTCTGGGGCTTCAAGTCCGACTT
CAGCCATTTCGCATCGCCCCAGGGCGTGTCGCGTTTCATCACCGGTGACTATGGTCTGGG
CCACACCGTGCTGCCTGCTCCCGACTTTGACAAGACCGTGGCCTTTGTGCGCAATGTGC
TGGGCTTTGGCCTCTCGGATATTTACAACTTCAAGCCCGCAGGCGATGCCGGCCCCTACGG
TCCGAATCCACTTCTTCCACTGCGCCAACGCTCGTCACCACAGCCTGGCGCTGGCCGAA
TTCCCTTCTCCCTCGGGTTGCGTGCACGTGATGGTGGAGGTGGACAACATGCCCGAAGT
GGGCCGTGCCATGGACCGCATGCAAAAGAGCCAGGTCAAGCTCTCTGCCACTCTGGGT
CAGCACACCAACGACAAGATGATCTCGTTCTACATGAAGACGCCCTCCAACTTCGACCT
GGAGTTCGGCTATGGCGGCGCCATCATCGACTGGGAACACCACATCACGCACGAGTTCA
CGACCGTAAGCCTCTGGGGGCACGACTTT

>Aerobic DHSTP *tesB*_4
TTGGCATCCGGATGGGAGGTGGCCAACAAGGAAGCCTTCGAGCAGGCGCTGAAGGTGC
TGCAGGCTGCCGACGTGCACTACGAGATGGGCAGCCCCGAGCTATGCGCCAAGCGCCAT
GTGCAGCAGCTGGTCATCGTGATGGATCCATCGGGCAACCGCCACGAGATCGTCTGGGG
CTTCAAGTCCGACTTCACTCACTTTGCCTCGCCCCAAGGGGTGTCGCGCTTCATCACCG
GCGACTACGGCCTGGGCCATACCGTGCTGCCGGCGCCCGAGTTCGACAAGACCGTGGC
TTTTGCGCGCGACGTGCTGGGTTTCGACCTTTCTGACATCTACAACTTCAAGCCTGCGG
GCGATGCCGGTCCCACGATCCGCATCCACTTCTTCCACTGCGCCAACGGCCGCCACCAC
AGCCTGGCGCTGGCCGAGTTCCCTTCTGCATCGGGCTGCGTGCACGTGATGGTGGAGGT
GGACAACATGCCCGAGGTGGGCCGCGCCATGGACCGCATGCAAAAGAGCCAGGTCAAG
CTGTCCGCCACCCTGGGCCAGCACACCAACGACAAGATGATTTCGTTCTACATGAAGAC

GCCCTCGAACTTCGATCTGGAGTTCGGCTATGGCGGCGCCATCGTCGACTGGGGTCAGC
ACATCACGCATGAGTTCACGACCGTAAGCCT

>Aerobic DHSTP *tesB*_5
AGGTGGCCAACAAGGAAGCCTTCGAGCAGGCGCTGAAGGTGCTGCAGGCTGCCGACG
TGCACTACGAGATGGGCAGCCCCGAGCTATGCGCCAAGCGCCATGTGCAGCAGCTGGTC
ATCGTGATGGATCCATCGGGCAACCGCCACGAGATCGTCTGGGGCTTCAAGTCCGACTT
CACTCACTTTGCCTCGCCCCAAGGGGTGTCGCGTTTCATCACCGGCGACTACGGCCTGG
GCCATACCGTGCTGCCGGCGCCCGAGTTCGACAAGACCGTGGCTTTTGCGCGCGACGTG
CTGGGTTTCGACCTTTCTGACATCTACAACTTCAAGCCTGCGGGCGATGCCGGTCCCAC
GATCCGCATCCACTTCTTCCACTGCGCCAACGGCCGCCACCACAGCCTGGCGCTGGCCG
AGTTCCCTTCTGCATCGGGCTGCGTGCACGTGATGGTGGAGGTGGACAACATGCCCGAG
GTGGGCCGCGCCATGGACCGCATGCAAAAGAGCCAGGTCAAGCTGTCCGCCACCCTGG
GCCAGCACACCAACGACAAGATGATTTCGTTCTACATGAAGACGCCCTCGAACTTCGAT
CTGGAGTTCGGCTATGGCGGCGCCATCGTCGACTGGGATCAGCACATCACGCATGAGTT
CACGACCGTAAGCCTCTGGGGGCACGACTT

>Aerobic DHSTP *tesB*_6
ATTCCGCATCTGGTTGGGAGGTGGCCAACAAGGAAGCCTTTGAGCAGGCGCTCAAGGT
GCTGCAGGCTGCCGACGTGCACTACGAGATGGGCAGCCCCGAGCTGTGCGCCAAGCGC
CATGTGCAGCAGCTGGTCATAGTGATGGATCCATCGGGCAACCGCCACGAGATCGTCTG
GGGCTTCAAGTCCGACTTCACTCACTTTGCCTCGCCCCAAGGGGTGTCGCGCTTCATCA
CCGGCGACTACGGCCTGGGCCATACCGTGCTGCCGGCGCCCGAGTTCGACAAGACCGT
GGCTTTTGCGCGCGACGTGCTGGGTTTCGACCTTTCTGACATCTACAACTTCAAGCCTGC
GGGCTATGCCGGTCCCACGATCCGCATCCACTTCTTCCACTGCGCCAACGGCCGCCACC
ACAGCCTGGCGCTGGCGGAGTTCCCCGCGGCCAGCGGCTGCGTGCACGTGATGGTCGA
GGTGGACAACATGCCCGAGGTCGGCCGCGCCATGGACCGCATGCAAAAGAGCCAGGTC
AAGCTGTCCGCCACCCTGGGCCAGCACACCAACGACAAGATGATTTCGTTCTACATGAA
GACGCCCTCGAACTTCGATCTGGAGTTCGGCTATGGCGGCGCCATCGTCGACTGGGATC
AGCACATCACGCATGAGTTCACAACCGTAAGCCTC

>Aerobic DHSTP *tesB*_7
ACCCCGCATCCGGCTGGGAGGTGGCCAACAAGCAAGCCTTCGAGCAGGCGCTCGAGGT
GTTGCAGGCCGCTGATGTGCACTACGAAATGGGCCGCCCCGAGCTGTGTGCCAAGCGCC
ATGTGCAGGAGTTGGCCATCGTGATGGATCCGTCCGGAAACCGTCATGAGATCGTCTGG
GGCTTCAAGTCCGACTTCAGCCATTTCGCATCGCCCCAGGGCGTGTCGCGTTTCATCACC
GGTGACTATGGTCTGGGCCACACCGTGCTGCCTGCTCCCGACTTTGACAAGACCGTGGC
CTTTGTGCGCAATGTGCTGGGCTTTGGCCTCTCGGATATTTACAACTTCAAGCCCGCAGG
CGATGCCGGCCCTACGGTCCGAATCCACTTCTTCCACTGCGCCAACGCTCGCCACCACA
GCCTGGCGCTGGCCGAATTCCCTTCTCCCTCGGGTTGCGTGCACGTGATGGTGGAGGTG
GACAACATGCCCGAAGTGGGCCGTGCCATGGACCGCATGCAAAAGAGCCAGGTCAAGC
TCTCTGCCACTCTGGGTCAGCACACCAACGACAAGATGATCTCGTTCTACATGAAGACG
CCCTCCAACTTCGACCTGGAGTTCGGCTATGGCGGCGCCATCATCGACTGGGAACACCA
CATCACGCACGAGTTCACGACCGTAAGCC

>Aerobic DHSTP *tesB*_8
TATCCGGCCTCGGGTTGGGAGGTGGCCAACAAGGAAGCCTTTGAGCAGGCGCTCAAGG
TGCTGCAGGCTGCCGATGTGCACTACGAGATGGGCAGCCCCGAGCTGTGCGCCAAGCG
CCATGTGCAGCAACTGGTCATCGTGATAGATCCATCGGGCAATCGCCACGAGATCGTCTG
GGGCTTCAAGTCCGATTTCACTCACTTTGCCTCGCCTCAAGGGGTGTCGCGCTTCATCAC
CGGCGACTACGGCCTGGGCCATACCGTGCTGCCGGCGCCCGAGTTCGACAAGACCGTG
AAATTTGCGCGCGACGTGCTGGGTTTCGGCCTGTCGGACATCTACAACTTCAAGCCTGC
GGGCGATGCCGGTCCCACGATCCGCATCCACTTCTTCCACTGCGCCAACGGCCGCCACC
ACAGCCTGGCGCTGGCCGAGTTCCCTTCTCCATCGGGCTGCGTGCACGTGATGGTCGAG
GTGGACAACATGCCCGAGGTCGGCCGCGCCATGGACCGCATGCAAAAGAGCCAGGTCA
AGCTCTCCGCCACCCTGGGCCAGCACACCAACGACAAGATGATTTCGTTCTACATGAAG
ACGCCCTCGAACTTCGATCTGGAGTTCGGCTATGGCGGCGCCATCGTCGACTGGGATCA

GCACATCACGCATGAGTTCACGACCGTAAGCCT

>Aerobic DHSTP *tesB*_9
TTGGCATCTGGTTGGGAGGTGGCCAACAAGCAAGCCTTCGAGCAGGCGCTCGAGGTGT
TGCAGGCCGCTGATGTGCACTACGAAATGGGCCGCCCCGAGCTGTGTGCCAAGCGCCAT
GTGCAGGAGTTGGCCATCGTGATGGATCCGTCCGGAAACCGTCATGAGATCGTCTGGGG
CTTCAAGTCCGACTTCAGCCATTTCGCATCGCCTCAGGGCGTGTCGCGTTTCATCACCGG
TGACTATGGTCTGGGCCACACCGTGCTGCCTGCTCCCGACTTTGACAAGACCGTGGCCT
TTGTGCGCAATGTGCTGGGCTTTGGCCTCTCGGATATTTACAACTTCAAGCCCGCAGGCG
ATGCCGGCCCTACGGTCCGAATCCACTTCTTCCACTGCGCCAACGCTCGTCACCACAGC
CTGGCGCTGGCCGAATTCCCTTCTCCCTCGGGTTGCGTGCACGTGATGGTGGAGGTGGA
CAACATGCCCGAAGTGGGCCGTGCCATGGACCGCATGCAAAAGAGCCAGGTCAAGCTC
TCTGCCACTCTGGGTCAGCACACCAACGACAAGATGATCTCGTTCTACATGAAGACGCC
CTCCAACTTCGACCTGGAGTTCGGCTATGGCGGCGCCATCATCGACTGGGAACACCACA
TCACGCACGAGTTCACGACCGTAAGCCTC

>Aerobic DHSTP *tesB*_10&12
GGAGGTGGCCAACAAGGAAGCCTTCGAGCAGGCGCTGAAGGTGCTGCAGGCTGCCGA
CGTGCACTACGAGATGGGCAGCCCCGAGCTATGCGCCAAGCGCCATGTGCAGCAGCTG
GTCATCGTGATGGATCCATCGGGCAACCGCCACGAGATCGTCTGGGGCTTCAAGTCCGA
CTTCACTCACTTTGCCTCGCCCCAAGGGGTGTCGCGCTTCATCACCGGCGACTACGGCC
TGGGCCATACCGTGCTGCCGGCGCCCGAGTTCGACAAGACCGTGGCTTTTGCGCGCGAC
GTGCTGGGTTTCGACCTTTCTGACATCTACAACTTCAAGCCTGCGGGCGATGCCGGTCC
CACGATCCGCATCCACTTCTTCCACTGCGCCAACGGCCGCCACCACAGCCTGGCGCTGG
CCGAGTTCCCTTCTGCATCGGGCTGCGTGCACGTGATGGTGGAGGTGGACAACATGCCC
GAGGTGGGCCGCGCCATGGACCGCATGCAAAAGAGCCAGGTCAAGCTGTCCGCCACCC
TGGGCCAGCACACCAACGACAAGATGATTTCGTTCTACATGAAGACGCCCTCGAACTTC
GATCTGGAGTTCGGCTATGGCGGCGCCATCGTCGACTGGGATCAGCACATCACGCATGA
GTTCACGACCGTAAGCCTCTGGGGCCACGACTT

>Aerobic DHSTP *tesB*_11
CCCGCCTCTGGATGGGAAGTGGCCAACAAGCACGCCTTTGAGCAGGCGCTCAAGGTGT
TGCAGGCAGCTGATGTGCACTACGAAATGGGCAGTTCCGAACTGTGTGCCAAGCGCCAT
GTGCAGGAGCTGGCCATCGTGATGGATCCGTCCGGAAATTGTCACGAGATCGTCTGGGG
CTTCAAGTCCGACTTCCGCCATTTTGCGTCGCCCCAAGGGGTGTCGCGCTTTATCACCGG
CGACTATGGTCTGGGCCACACCGTGCTGCCCGCTCCCGACTTTGACAAGACCGTGGCCT
CTGTGCGCAATGTGCTGGGCTTTGGTCTCTCGGATATTTACAACTTCAAGCCCGCAGGCG
ATGCCGGCCCCACGGTCCGAATCCACTTCTTCCACTGCGCCAACGGGCGTCACCACAGC
CTGGCGCTGGCCGAGTTCCCTTCCGCATCGGGTTGCGTGCACGTGATGGTGGAGGTGGA
CAACATGCCCGAAGTGGGCCGTGCCATGGACCGCATGCAAAAGAGCCAGGTCAAGCTC
TCTGCCACCCTGGGTCAGCACACCAACGACAAGATGATCTCGTTCTACATGAAGACGCC
CTCCAACTTCGATCTGGAGTTCGGCTATGGCGGCGCCGTCATCGACTGGGAGCACCACA
TCACGCACGAGTTCACGACCGTAAGCCTC

>Aerobic DHSTP *tesB*_13
TTTATCTCGTATCTGGATGGGAGGTGGCCAACAAGGAAGCCTTTGAGCAGGCGCTCAAG
GTGCTGCAGGCTGCCGACGTGCACTACGAGATGGGCAGCCCCGAGCTGTGCGCCAAGC
GCCATGTGCAGCAGCTGGTCATAGTGATGGATCCATCGGGCAACCGCCACGAGATCGTC
TGGGGCTTCAAGTCCGACTTCACTCACTTTGCCTCGCCCCAAGGGGTGTCGCGCTTCAT
CACCGGCGACTACGGCCTGGGCCATACCGTGCTGCCGGCGCCCGAGTTCGACAAGACC
GTGGCTTTTGCGCGCGACGTGCTGGGTTTCGACCTTTCTGACATCTACAACTTCAAGCCT
GCGGGCTATGCCGGTCCCACGATCCGCATCCACTTCTTCCACTGCGCCAACGGCCGCCA
CCACAGCCTGGCGCTGGCGGAGTTCCCCGCGGCCAGCGGCTGCGTGCACGTGATGGTC
GGGGTGGACAACATGCCCGAGGTCGGCCGCGCCATGGACCGCATGCAAAAGAGCCAGG
TCAAGCTGTCCGCCACCCTGGGCCAGCACACCAACGACAAGATGATTTCGTTCTACATG
AAGACGCCCTCGAACTTCGATCTGGAGTTCGGCTATGGCGGCGCCATCGTCGACTGGGA
TCAGCACATCACGCATGAGTTCACAACCGTAAGCCTCT

>Aerobic DHSTP *tesB*_14
ATTTGGCATCTGGTTGGGAGGTGGCCAACAAGCAAGCCTTCGAGCAGGCGCTCGAGGT
GTTGCAGGCCGCTGATGTGCACTACGAAATGGGCCGCCCCGAGCTGTGTGCCAAGCGCC
ATGTGCAGGAGTTGGCCATCGTGATGGATCCGTCCGGAAACCGTCATGAGATCGTCTGG
GGCTTCAAGTCCGACTTCAGCCATTTCGCATCGCCTCAGGGCGTGTCGCGTTTCATCACC
GGTGACTATGGTCTGGGCCACACCGTGCTGCCTGCTCCCGACTTTGACAAGACCGTGGC
CTTTGTGCGCAATGTGCTGGGCTTTGGCCTCTCGGATATTTACAACTTCAAGCCCGCAGG
CGATGCCGGCCCTACGGTCCGAATCCACTTCTTCCACTGCGCCAACGCTCGTCACCACA
GCCTGGCGCTGGCCGAATTCCCTTCTCCCTCGGGTTGCGTGCACGTGATGGTGGAGGTG
GACAACATGCCCGAAGTGGGCCGTGCCATGGACCGCATGCAAAAGAGCCAGGTCAAGC
TCTCTGCCACTCTGGGTCAGCACACCAACGACAAGATGATCTCGTTCTACATGAAGACG
CCCTCCAACTTCGACCTGGAGTTCGGCTATGGCGGCGCCATCATCGACTGGGAACACCA
CATCACGCACGAGTTCACGACCGTAAGCCTC

>Aerobic DHSTP *tesB*_15
GGAAGTGGCCAACAAGCAAGCCTTTGGGCAGGCGCTCAAGGTGTTGCAGGCAGCTGAT
GTGCACTACGAAATGGGCAGTTCCGAACTGTGTGCCAAGCGCCATGTGCAGGAGCTGG
CCATCGTGATGGATCCGTCCGGAAATCGTCACGAGATCGTCTGGGGCTTCAAGTCCGAC
TTCCGCCATTTTGCGTCGCCCCAAGGGGTGTCGCGCTTTATCACCGGCGACTATGGTCTG
GGCCACACCGTGCTGCCCGCTCCCGACTTTGACAAGACCGTGGCCTTTGTGCGCAATGT
GCTGGGCTTTGGTCTCTCGGATATTTACAACTTCAAGCCCGCAGGCGATGCCGGCCCCA
CGGTCCGAATCCACTTCTTCCACTGCGCCAACGGGCGTCACCACAGCCTGGCGCTGGCC
GAGTTCCCTTCCGCATCGGGTTGCGTGCACGTGATGGTGGAGGTGGACAACATGCCCGA
AGTGGGCCGTGCCATGGACCGCATGCAAAAGAGCCAGGTCAAGCTCTCTGCCACCCTG
GGTCAGCACACCAACGACAAGATGATCTCGTTCTATATGAAGACGCCCTCCAACTTCGA
CCTGGAGTTCGGCTATGGCGGCGCCGTCATCGACTGGGAGCACCACATCACGCACGAGT
TCACGACCGTAAGCCTCTGGGGCCACGACTTC

>Aerobic DHSTP *tesB*_16
GCATCTGGATGGGAGGTGGCCAACAAGCAAGCCTTCGAGCAGGCGCTCGAGGTGTTGC
AGGCCGCTGATGTGCACTACGAAATGGGCCGCCCCGAGCTGTGTGCCAAGCGCCATGTG
CAGGAGTTGGCCATCGTGATGGATCCGTCCGGAAACCGTCATGAGATCGTCTGGGGCTT
CAAGTCCGACTTCAGCCATTTCGCATCGCCCCAGGGCGTGTCGCGTTTCATCACCGGTG
ACTATGGTCTGGGCCACACCGTGCTGCCTGCTCCCGACTTTGACAAGACCGTGGCCTTT
GTGCGCAATGTGCTGGGCTTTGGCCTCTCGGATATTTACAACTTCAAGCCCGCAGGCGAT
GCCGGCCCTACGGTCCGAATCCACTTCTTCCACTGCGCCAACGCTCGTCACCACAGCCT
GGCGCTGGCCGAATTCCCTTCTCCCTCGGGTTGCGTGCACGTGATGGTGGAGGTGGACA
ACATGCCCGAAGTGGGCCGTGCCATGGACCGCATGCAAAAGAGCCAGGTCAAGCTCTC
TGCCACTCTGGGTCAGCACACCAACGACAAGATGATCTCGTTCTACATGAAGACGCCCT
CCAACTTCGACCTGGAGTTCGGCTATGGCGGCGCCATCATCGACTGGGAACACCACATC
ACGCACGAGTTCACGACCGTAAGCC

>Aerobic DHSTP *tesB*_17
TTCGGCATCGGGATGGGAAGTGGCCAACAAGCAAGCCTTTGAGCAGGCGCTCAAGGTG
TTGCAGGCAGCTGATGTGCACTACGAAATGGGCAGTTCCGAACTGTGTGCCAAGCGCCA
TGTGCAGGAGCTGGCCATCGTGATGGATCCGTCCGGAAATCGTCACGAGATCGTCTGGG
GCTTCAAGTCCGACTTCCGCCATTTTGCGTCGCCCCAAGGGGTGTCGCGCTTTATCACCG
GCGACTATGGTCTGGGCCACACCGTGCTGCCCGCTCCCGACTTTGACAAGACCGTGGCC
TTTGTGCGCAATGTGCTGGGCTTTGGTCTCTCGGATATTTACAACTTCAAGCCCGCAGGC
GATGCCGGCCCCACGGTCCGAATCCACTTCTTCCACTGCGCCAACGGGCGTCACCACAG
CCTGGCGCTGGCCGAGTTCCCTTCCGCATCGGGTTGCGTGCACGTGATGGTGGAGGTGG
ACAACATGCCCGAAGTGGGCCGTGCCATGGACCGCATGCAAAAGAGCCAGGTCAAGCT
CTCTGCCACCCTGGGTCAGCACACCAACGACAAGATGATCTCGTTCTACATGAAGACGC
CCTCCAACTTCGATCCGGAGTTCGGCTATGGCGGCGCCGTCATCGACTGGGAGCACCAC
ATCACGCACGAGTTCACGACCGTAAGCCT

>Aerobic DHSTP *tesB*_18
TTCGGCATTGGGATGGGAAGTGGCCAACAAGCAAGCCTTTGAGCAGGCGCTCAAGGTG
CTGCAGGCAGCTGATGTGCACTACGAAATGGGCAGCTCCGAGCTGTGTGCCAAGCGCCA
TGTGCAGGAGCTGGCCATCGTGGTGGATCCGTCCGGAAACCGTCATGAGATCGTCTGGG
GCTTCAAGTCCGACTTCAGCCATTTCGCATCGCCCCAGGGGGTTTCACGCTTCGTCACC
GGCGACTATGGCCTGGGCCATACCGTGCTGCCCGCTCCCGACTTCGACAAGACCGTGGC
CTTTGTGCGCAATGTACTGGGCTTTGGTCTTTCGGACATCTACAACTTCAAGCCCGCAGG
CGATGCCGGCCCCACGGTCCGCATTCATTTCTTCCACTGCGCCAACGCTCGTCACCACA
GTCTGGCGCTGGCCGAGTTCCCTTCCGCTTCGGGCTGCGTGCATGTGATGGTGGAGGTG
GACAACATGCCCGAAGTGGGCCGTGCCATGGACCGCATGCAAAAGAGCCAGGTCAAGC
TCTCTGCCACTCTGGGTCAGCACACCAACGACAAGATGATCTCGTTCTACATGAAGACG
CCCTCCAACTTCGACCTGGAGTTCGGCTATGGCGGTGCCGTCATCGACTGGGAACACCA
CATCGCGCACGAGTTCACGACCGTAAGCCTC

>Aerobic DHSTP *tesB*_19
ATCCCGCATCTGGATGGGAGGTGGCCAACAAGCAAGCCTTCGAGCAGGCGCTCGAGGT
GTTGCAGGCCGCTGATGTGCACTACGAAATGGGCCGCCCCGAGCTGTGTGCCAAGCGCC
ATGTGCAGGAGTTGGCCATCGTGATGGATCCGTCCGGAAACCGTCATGAGATCGTCTGG
GGCTTCAAGTCCGACTTCAGCCATTTCGCATCGCCCCAGGGCGTGTCGCGTTTCATCACC
GGTGACTATGGTCTGGGCCACACCGTGCTGCCTGCTCCCGACTTTGACAAGACCGTGGC
CTTTGTGCGCAATGTGCTGGGCTTTGGCCTCTCGGATATTTACAACTTCAAGCCCGCAGG
CGATGCCGGCCCTACGGTCCGAATCCACTTCTTCCACTGCGCCAACGCTCGTCACCACA
GCCTGGCGCTGGCCGAATTCCCTTCTCCCTCGGGTTGCGTGCACGTGATGGTGGAGGTG
GACAACATGCCCGAAGTGGGCCGTGCCATGGACCGCATGCAAAAGAGCCAGGTCAAGC
TCTCTGCCACTCTGGGTCAGCACACCAACGACAAGATGATCTCGTTCTACATGAAGACG
CCCTCCAACTTCGACCTGGAGTTCGGCTATGGCGGCGCCATCATCGACTGGGAACACCA
CATCACGCACGAGTTCACGACCGTAAGCCTC

>Aerobic DHSTP *tesB*_20
AGGTGGCCAACAAGGAAGCCTTCGAGCAGGCGCTGAAGGTGCTGCAGGCTGCCGACG
TGCACTACGAGATGGGCAGCCCCGAGCTATGCGCCAAGCGCCATGTGCAGCAGCTGGTC
ATCGTGATGGATCCATCGGGCAACCGCCACGAGATCGTCTGGGGCTTCAAGTCCGACTT
CACTCACTTTGCCTCGCCCCAAGGGGTGTCGCGCTTCATCACCGGCGACTACGGCCTGG
GCCATACCGTGCTGCCGGCGCCCGAGTTCGACAAGACCGTGGCTTTTGCGCGCGACGTG
CTGGGTTTCGACCTTTCTGACATCTACAACTTCAAGCCTGCGGGCGATGCCGGTCCCAC
GATCCGCATCCACTTCTTCCACTGCGCCAACGGCCGCCACCACAGCCTGGCGCTGGCCG
AGTTCCCTTCTGCATCGGGCTGCGTGCACGTGATGGTGGAGGTGGACAACATGCCCGAG
GTGGGCCGCGCCATGGACCGCATGCAAAAGAGCCAGGTCAAGCTGTCCGCCACCCTGG
GCCAGCACACCAACGACAAGATGATTTCGTTCTACATGAAGACGCCCTCGAACTTCGAT
CTGGAGTTCGGCTATGGCGGCGCCATCGTCGACTGGGATCAGCACATCACGCATGAGTT
CACGACCGTAAGCCTCTGGGGCCACGACTTC

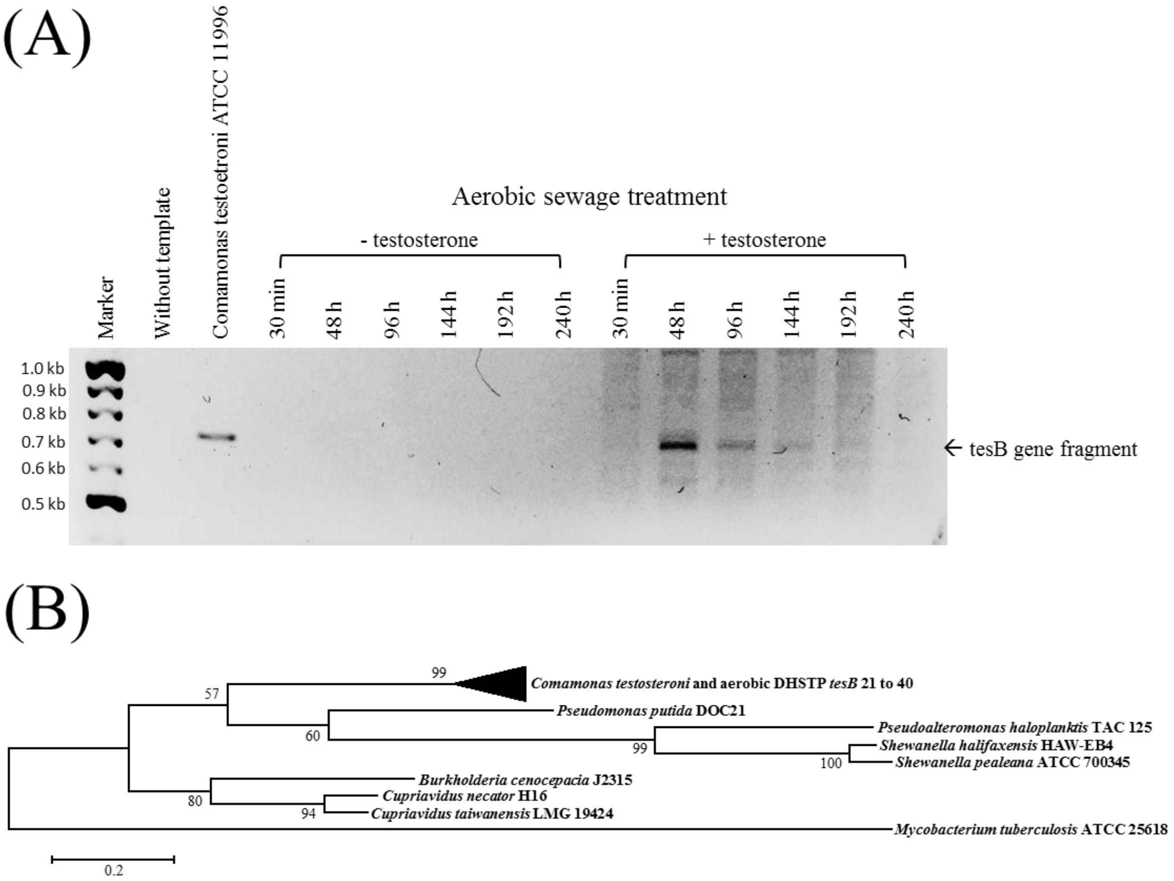

Figure S7. PCR-based functional assay using the DNA extracted from testosterone-amended sewage Replicate 2 as the templates. The degenerate primers (*tesB*-f1/*tesB*-r1) used in the assay were derived from eight proteobacterial *tesB* genes. (A) The *tesB*-like PCR products increased only in the aerobic sewage incubated with testosterone. (B) The phylogenetic tree of the *tesB* genes obtained from aerobic sewage incubated with testosterone for 48 hours. Refer to Fig. S7 for the *tesB* sequences amplified from the sewage Replicate 2. The sequence of gene encoding 3,4-DHSA dioxygenase from *M. tuberculosis* ATCC 25618 served as an outgroup sequence.

Figure S8. Twenty *tesB* DNA fragments obtained from another testosterone-amended sewage replicate (incubated with testosterone for 48 hours ; Replicate 2) by using the degenerate primers *tesB*-f1/*tesB*-r1.

>Aerobic DHSTP *tesB*_21
GGTGGCCAACAAGCAAGCCTTCGAGCAGGCGCTCGAGGTGTTGCAGGCCGCTGATGTG
CACTACGAAATGGGCCGCCCCGAGCTGTGTGCCAAGCGCCATGTGCAGGAGTTGGCCAT
CGTGATGGATCCGTCCGGAAACCGTCATGAGATCGTCTGGGGCTTCAAGTCCGACTTCA
GCCATTTCGCATCGCCCCAGGGCGTGTCGCGTTTCATCACCGGTGACTATGGTCTGGGCC
ACACCGTGCTGCCTGCTCCCGACTTTGACAAGACCGTGGCCTTTGTGCGCAATGTGCTG
GGCTTTGGCCTCTCGGATATTTACAACTTCAAGCCCGCAGGCGATGCCGGCCCTACGGTC
CGAATCCACTTCTTCCACTGCGCCAACGCTCGTCACCACAGCCTGGCGCTGGCCGAATT
CCCTTCTCCCTCGGGTTGCGTGCACGTGATGGTGGAGGTGGACAACATGCCCGAAGTGG
GCCGTGCCATGGACCGCATGCAAAAGAGCCAGGTCAAGCTCTCTGCCACTCTGGGTCA
GCACACCAACGACAAGATGATCTCGTTCTACATGAAGACGCCCTCCAACTTCGACCTGG
AGTTCGGCTATGGCGGCGCCATCATCGACTGGGAACACCACATCACGCACGAGTTCACG
ACCGTAAGCCTC

>Aerobic DHSTP *tesB*_22
AGTGGCCAACAAGGAAGCTTTTGACAAAGCGCTGAAGGTACTGGAGGCGGCTGACGTG
CGCTATGAAATGGGCAGCGCCGAGCTGTGCACCAAGCGCCGCGTGCAGCAACTGGCGG
TGGTGATCGATCCTTCGGGCAACCGCCACGAAATCGTCTGGGGCTTCAAGTCCGATTTC
ATCCACTTTGCCTCGCCGCAAGGCGTTTCCCGCTTCATCACGGGTGACTACGGCCTGGG
CCACACCGTGCTGCCCGCTCCCGACTTTGAAAAGACCGTGGCTTTTGTGCGCAATGTCC
TGGGTTTTGGTCTGTCGGACATCTACAACTTCAAGCCCGCTGGTGATGCAGGCCCCACG
GTTCGCATTCACTTCTTCCACTGCGCCAATGGCCGCCACCACAGTCTGGCGCTGGCCGA
GTTCCCCTCGCCCACAGGCTGCGTGCACGTGATGGTGGAAGTGGACAACATGCCCGAA
GTGGGCCGTGCCATGGACCGCATGCAAAAGAGCCAGACCAAGCTGTCGGCCACGCTGG
GCCAGCACACCAACGACAAGATGATTTCCTTCTACATGAAGACGCCCTCGAACTTCGAT
CTGGAGTTTGGCTACGGCGGCGCAATCATCGACTGGGATCACCACATCACCCACGAATT
CACGACCGTAAGTCTC

>Aerobic DHSTP *tesB*_23
GGTGGCCAACAAGCAAGCCTTCGAGCAGGCGCTCAAGGTGCTGCAGGCCGCTGATGTG
CACTACGAAATGGGCAGTTCCGAACTGTGTGCCAAGCGCCATGTGCAGGAGTTGGCCAT
CGTGATGGATCCGTCCGGAAACCGTCATGAGATCGTCTGGGGCTTCAAGTCCGACTTCA
GCCATTTCGCATCGCCCCAGGGTGTTTCGCGCTTCATCACCGGTGACTATGGTCTGGGCC
ACACCGTGCTGCCTGCTCCCGACTTTGACAAGACCGTGGCCTTTGTGCGCAATGTGCTG
GGCTTTGGCCTCTCGGATATTTACAACTTCAAGCCCGCAGGCGATGCCGGCCCCACGGT
CCGAATCCACTTCTTCCACTGCGCCAACGCTCGTCACCACAGCCTGGCTCTGGCCGAAT
TTCCTTCTCCCTCGGGTTGCGTGCACGTGATGGTGGAGGTGGACAACATGCCCGAAGTG
GGTCGTGCCATGGACCGCATGCAAAAGAGCCAGGTCAAGCTCTCGGCCACTCTGGGTC
AGCACACCAACGACAAGATGATCTCGTTCTACATGAAGACGCCCTCCAACTTCGACCTG
GAGTTCGGCTATGGCGGCGCCATCATCGACTGGGAACATCACATCACGCACGAGTTCAC
GACCGTAAGCCTC

>Aerobic DHSTP *tesB*_24
AGTGGCCAACAAGGAAGCTTTTGACAAAGCGCTGAAGGTACTGGAGGCGGCTGACGTG
CGCTATGAAATGGGCAGCGCCGAGCTGTGCACCAAGCGCCGCGTGCAGCAACTGGCGG
TGGTGATCGATCCTTCGGGCAACCGCCACGAAATCGTCTGGGGCTTCAAGTCCGATTTC
ATCCACTTTGCCTCGCCGCAAGGCGTTTCCCGCTTCATCACGGGTGACTACGGCCTGGG
CCACACCGTGCTGCCCGCTCCCGACTTTGAAAAGACCGTGGCTTTTGTGCGCAATGTCC
TGGGTTTTGGTCTGTCGGACATCTACAACTTCAAGCCCGCTGGTGATGCAGGCCCCACG
GTTCGCATTCACTTCTTCCACTGCGCCAATGGCCGCCACCACAGTCTGGCGCTGGCCGA
GTTCCCCTCGCCCACAGGCTGCGTGCACGTGATGGTGGAAGTGGACAACATGCCCGAA
GTGGGCCGTGCCATGGACCGCATGCAAAAGAGCCAGACCAAGCTGTCGGCCACGCTGG

GCCAGCACACCAACGACAAGATGATTTCCTTCTACATGAAGACGCCCTCGAACTTCGAT
CTGGAGTTTGGCTACGGCGGCGCAATCATCGACTGGGATCACCACATCACCCACGAATT
CACGACCGTAAGTCTC

>Aerobic DHSTP *tesB*_25
GGTGGCCAACAAGGAAGCCTTCGAGCAGGCGCTGAAGGTGCTGCAGGCTGCCGACGT
GCACTACGAGATGGGCAGCCCCGAGCTATGCGCCAAGCGCCATGTGCAGCAGCTGGTCA
TCGTGATGGATCCATCGGGCAACCGCCACGAGATCGTCTGGGGCTTCAAGTCCGACTTC
ACTCACTTTGCCTCGCCCCAAGGGGTGTCGCGCTTCATCACCGGCGACTACGGCCTGGG
CCATACCGTGCTGCCGGCGCCCGAGTTCGACAAGACCGTGGCTTTTGCGCGCGACGTGC
TGGGTTTCGACCTTTCTGACATCTACAACTCCGAGCCTGCGGGCGATGCCGGTCCCACG
ATCCGCATCCACTTCTTCCACTGCGCCAACGGCCGCCACCACAGCCTGGCGCTGGCCGA
GTTCCCTTCTGCATCGGGCTGCGTGCACGTGATGGTGGAGGTGGACAACATGCCCGAGG
TGGGCCGCGCCATGGACCGCATGCAAAAGAGCCGGGTCAAGCTGTCCGCCACCCTGGG
CCAGCACACCAACGACAAGATGATTTCGTTCTACATGAAGACGCCCTCGAACTTCGATC
TGGAGTTCGGCTATGGCGGCGCCATCGTCGACTGGGATCAGCACATCACGCATGAGTTC
ACGACCGTAAGCCTC

>Aerobic DHSTP *tesB*_26
AGTGGCCAACAAGGAAGCTTTTGACAAAGCGCTGAAGGTACTGGAGGCGGCTGACGTG
CGCTATGAAATGGGCAGCGCCGAGCTGTGCACCAAGCGCCGCGTGCAGCAACTGGCGG
TGGTGATCGATCCTTCGGGCAACCGCCACGAAATCGTCTGGGGCTTCAAGTCCGATTTC
ATCCACTTTGCCTCGCCGCAAGGCGTTTCCCGCTTCATCACGGGTGACTACGGCCTGGG
CCACACCGTGCTGCCCGCTCCCGACTTTGAAAAGACCGTGGCTTTTGTGCGCAATGTCC
TGGGTTTTGGTCTGTCGGACATCTACAACTTCAAGCCCGCTGGTGATGCAGGCCCCACG
GTTCGCATTCACTTCTTCCACTGCGCCAATGGCCGCCACCACAGTCTGGCGCTGGCCGA
GTTCCCCTCGCCCACAGGCTGCGTGCACGTGATGGTGGAAGTGGACAACATGCCCGAA
GTGGGCCGTGCCATGGACCGCATGCAGAAGAGCCAGACCGAGCTGTCGGCCACGCTGG
GCCAGCACACCAACGACAAGATGATTTCCTTCTACATGAAGACGCCCTCGAACTTCGAT
CCGGAGTTTGGCTACGGCGGCGCAATCATCGACTGGGATCACCACATCACCCACGAATT
CACGACCGTAAGTCTC

>Aerobic DHSTP *tesB*_27
GGTGGCCAACAAGGAAGCCTTCGAGCAGGCGCTGAAGGTGCTGCAGGCTGCCGACGT
GCACTACGAGATGGGCAGCCCCGAGCTATGCGCCAAGCGCCATGTGCAGCAGCTGGTCA
TCGTGATGGATCCATCGGGCAACCGCCACGAGATCGTCTGGGGCTTCAAGTCCGACTTC
ACTCACTCTGCCTCGCCCCAAGGGGTGTCGCGCTTCATCACCGGCGACTACGGCCTGGG
CCATACCGTGCTGCCGGCGCCCGAGTTCGACAAGACCGTGGCTTTTGCGCGCGACGTGC
TGGGTTTCGACCTTTCTGACATCTACAACTTCAAGCCTGCGGGCGATGCCGGTCCCACG
ATCCGCATCCACTTCTTCCACTGCGCCAACGGCCGCCACCACAGCCTGGCGCTGGCCGA
GTTCCCTTCTGCATCGGGCTGCGTGCACGTGATGGTGGAGGTGGACAACATGCCCGAGG
TGGGCCGCGCCATGGACCGCATGCAAAAGAGCCAGGTCAAGCTGTCCGCCACCCTGGG
CCAGCACACCAACGACAAGATGATTTCGTTCTACATGAAGACGCCCTCGAACTTCGATC
TGGAGTTCGGCTATGGCGGCGCCATCGTCGACTGGGATCAGCACATCACGCATGAGTTC
ACGACCGTAAGCCTC

>Aerobic DHSTP *tesB*_28
AGTGGCCAACAAGCACGCCTTTGAGCAGGCGCTCAAGGTGTTGCAGGCAGCTGATGTG
CACTACGAAATGGGCAGTTCCGAACTGTGTGCCAAGCGCCATGTGCAGGAGCTGGCCAT
CGTGATGGATCCGTCCGGAAATCGTCACGAGATCGTCTGGGGCTTCAAGTCCGACTTCC
GCCATTTTGCGTCGCCCCAAGGGGTGTCGCGCTTTATCACCGGCGACTATGGTCTGGGCC
ACACCGTGCTGCCCGCTCCCGACTTTGACAAGACCGTGGCCTTTGTGCGCAATGTGCTG
GGCTTTGGTCTCTCGGATATTTACAACTTCAAGCCCGCAGGCGATGCCGGCCCCACGGT
CCGAATCCACTTCTTCCACTGCGCCAACGGGCGTCACCACAGCCTGGCGCTGGCCGAGT
TCCCTTCCGCATCGGGTTGCGTGCACGTGATGGTGGAGGTGGACAACATGCCCGAAGTG
GGCCGTGCCATGGACCGCATGCAAAAGAGCCAGGTCAAGCTCTCTGCCACCCTGGGTC
AGCACACCAACGACAAGATGATCTCGTTCTACATGAAGACGCCCTCCAACTTCGATCTG

GAATTCGGCTATGGCGGCGCCGTCATCGACTGGGAGCACCACATCACGCACGAGTTCAC
GACCGTAAGCCTC

>Aerobic DHSTP *tesB*_29
AGTGGCCAACAAGCAAGCCTTCGAGCAGGCGCTCGAGGTGTTGCAGGCCGCTGATGTG
CACTACGAAATGGGCAGCTCCGAACTGTGTGCCAAGCGCCATGTGCAGGAGTTGGCCAT
CGTGATGGATCCATCCGGAAACCGTCATGAGATCGTCTGGGGCTTCAAGTCCGACTTCA
GCCATTTCGCATCGCCCCAGGGTGTTTCGCGCTTCATCACCGGTGACTATGGTTTGGGTC
ACACCGTGCTGCCTGCTCCCGACTTTGACAAGACCGTGGCCTTTGTGCGCAATGTGCTG
GGCTTTGGCCTCTCGGATATTTACAACTTCAAGCCCGCAGGCGATGCCGGCCCCACGGT
CCGAATCCACTTCTTCCACTGCGCAAACGCTCGTCACCACAGCCTGGCGCTGGCCGAAT
TCCCTTCTCCCTCGGGTTGCGTGCACGTGATGGTGGAGGTGGACAACATGCCCGAAGTG
GGCCGTGCCATGGACCGCATGCAAAAGAGCCAGGTCAAGCTCTCGGCCACTCTGGGTC
AGCACACCAACGACAAGATGATCTCGTTCTACATGAAGACGCCCTCCAACTTCGACCTG
GAGTTCGGCTATGGCGGCGCCATCATCGACTGGGAACATCACATCACGCACGAGTTCAC
GACCGTAAGCCTC

>Aerobic DHSTP *tesB*_30
GGTGGCCAACAAGCAAGCCTTCGAGCAGGCGCTCGAGGTGTTGCAGGCCGCTGATGTG
CACTACGAAATGGGCCGCCCCGAGCTGTGTGCCAAGCGCCATGTGCAGGAGTTGGCCAT
CGTGATGGATCCGTCCGGAAACCGTCATGAGATCGTCTGGGGCTTCAAGTCCGACTTCA
GCCATTTCGCATCGCCCCAGGGCGTGTCGCGTTTCATCACCGGTGACTATGGTCTGGGCC
ACACCGTGCTGCCTGCTCCCGACTTTGACAAGACCGTGGCCTTTGTGCGCAATGTGCTG
GGCTTTGGCCTCTCGGATATTTACAACTTCAAGCCCGCAGGCGATGCCGGCCCTACGGTC
CGAATCCACTTCTTCCACTGCGCCAACGCTCGTCACCACAGCCTGGCGCTGGCCGAATT
CCCTTCTCCCCCGGGTTGCGTGCACGTGATGGTGGAGGTGGACAACATGCCCGAAGTGG
GCCGTGCCATGGACCGCATGCAAAAGAGCCAGGTCAAGCTCTCTGCCACTCTGGGTCA
GCACACCAACGACAAGATGATCTCGTTCTACATGAAGACGCCCTCCAACTTCGACCTGG
AGTTCGGCTATGGCGGCGCCATCATCGACTGGGAACACCACATCACGCACGAGTTCACG
ACCGTAAGCCTC

>Aerobic DHSTP *tesB*_31
GGTGGCCAACAAGGAAGCCTTCGAGCAGGCGCTGAAGGTGCTGCAGGCTGCCGACGT
GCACTACGAGATGGGCAGCCCCGAGCTATGCGCCAAGCGCCATGTGCAGCAGCTGGTCA
TCGTGATGGGTCCATCGGGCAACCGCCACGAGATCGTCTGGGGCTTCAAGTCCGACTTC
ACTCACTTTGCCTCGCCCCAAGGGGTGTCGCGCTTCATCACCGGCGACTACGGCCTGGG
CCATACCGTGCTGCCGGCGCCCGAGTTCGACAAGACCGTGGCTTTTGCGCGCGACGTGC
TGGGTTTCGACCTTTCTGACATCTACAACTTCAAGCCTGCGGGCGATGCCGGTCCCACG
ATCCGCATCCACTTCTTCCACTGCGCCAACGGCCGCCACCACAGCCTGGCGCTGGCCGA
GTTCCCTTCTGCATCGGGCTGCGTGCACGTGATGGTGGAGGTGGACAACATGCCCGAGG
TGGGCCGCGCCATGGACCGCATGCAAAAGAGCCAGGTCAAGCTGTCCGCCACCCTGGG
CCAGCACACCAACGACAAGATGATTTCGTTCTACATGAAGACGCCCTCGAACTTCGATC
TGGAGTTCGGCTATGGCGGCGCCATCGTCGACTGGGATCAGCACATCACGCATGAGTTC
ACGACCGTAAGCCTC

>Aerobic DHSTP *tesB*_32
GGTGGCCAACAAGGAAGCCTTTGAGCAGGCGCTCAAGGTGCTGCAGGCTGCCGATGTG
CACTACGAGATGGGCAGCCCCGAGCTGTGCGCCAAGCGCCATGTGCAGCAACTGGTCAT
CGTGATAGATCCATCGGGCAATCGCCACGAGATCGTCTGGGGCTTCAAGTCCGATTTCAC
TCACTTTGCCTCGCCTCAAGGGGTGTCGCGCTTCATCACCGGCGACTACGGCCTGGGCC
ATACCGTGCTGCCGGCGCCCGAGTTCGACAAGACCGTGAAATTTGCGCGCGACGTGCTG
GGTTTCGGCCTGTCGGACATCTACAACTTCAAGCCTGCGGGCGATGCCGGTCCCACGAT
CCGCATCCACTTCTTCCACTGCGCCAACGGCCGCCACCACAGCCTGGCGCTGGCCGAGT
TCCCTTCTCCATCGGGCTGCGTGCACGTGATGGTCGAGGTGGACAACATGCCCGAGGTG
GGCCGCGCCATGGACCGCATGCAAAAGAGCCAGGTCAAGCTCTCCGCCACTCTGGGCC
AGCACACCAACGACAAGATGATTTCGTTCTACATGAAGACGCCCTCGAACTTCGATCTG
GAGTTCGGCTATGGCGGCGCCATCGTCGACTGGGATCAGCACATCACGCATGAGTTCAC

GACCGTAAGCCTC

>Aerobic DHSTP *tesB*_33
AGTGGCCAACAAGCAAGCCTTTGGGCAGGCGCTCAAGGTGTTGCAGGCAGCTGATGTG
CACTACGAAATGGGCAGTTCCGAACTGTGTGCCAAGCGCCATGTGCAGGAGCTGGCCAT
CGTGATGGATCCGTCTGGAAACCGTCACGAGATCGTCTGGGGCTTCAAGTCCGACTTCC
GCCATTTTGCGTCGCCCCAAGGGGTGTCGCGCTTTATCACTGGCGATTATGGTCTGGGCC
ACACCGTGCTGCCTGCTCCCGACTTTGACAAGACCGTGGCCTTTGTACGCAATGTGCTG
GGCTTTGGTCTCTCGGATATTTACAACTTCAAGCCCGCAGGCGATGCCGGCCCCACGGT
CCGAATCCACTTCTTCCACTGCGCCAATGGGCGTCACCACAGCCTGGCGCTGGCCGAGT
TCCCTTCCGCATCGGGTTGCGTGCACGTGATGGTGGAGGTGGACAACATGCCCGAAGTG
GGCCGTGCCATGGACCGCATGCAAAAGAGCCAGGTCAAGCTCTCTGCCACCCTGGGTC
AGCACACCAGCGACAAGATGATCTCGTTCTACATGAAGACGCCCTCCAACTTCGACCTG
GAGTTCGGCTATGGCGGCGCCGTCATCGACTGGGAGCACCACATCACGCACGAGTTCAC
GACCGTAAGCCTC

>Aerobic DHSTP *tesB*_34
GGTGGCCAACAAGGAAGCCTTCGAGCAGGCGCTGAAGGTGCTGCAGGCTGCCGACGT
GCACTACGAGATGGGCAGCCCCGAGCTATGCGCCAAGCGCCATGTGCAGCAGCTGGTCA
TCGTGATGGATCCATCGGGCAACCGCCACGAGATCGTCTGGGGCTTCAAGTCCGACTTC
ACTCACTTTGCCTCGCCCCAAGGGGTGTCGCGCTTCATCACCGGCGACTACGGCCTGGG
CCATACCGTGCTGCCGGCGCCCGAGTTCGACAAGACCGTGGCTTTTGCGCGCGACGTGC
TGGGTTTCGACCTTTCTGACATCTACAACTTCAAGCCTGCGGGCGATGCCGGTCCCACG
ATCCGCATCCACTTCTTCCACTGCGCCAACGGCCGCCACCACAGCCTGGCGCTGGCCGA
GTTCCCTTCTGCATCGGGCTGCGTGCACGTGATGGTGGAGGTGGACAACATGCCCGAGG
TGGGCCGCGCCATGGACCGCATGCAAAAGAGCCAGGTCAAGCTGTCCGCCACCCTGGG
CCAGCACACCAACGACAAGATGATTTCGTTCTACATGAAGACGCCCTCGAACTTCGATC
TGGAGTTCGGCTATGGCGGCGCCATCGTCGACTGGGATCAGCACATCACGCATGAGTTC
ACGACCGTAAGCCTC

>Aerobic DHSTP *tesB*_35
GGTGGCCAACAAGGAAGCCTTCGAGCAGGCGCTGAAGGTGCTGCAGGCTGCCGACGT
GCACTACGAGATGGGCAGCCCCGAGCTATGCGCCAAGCACCATGTGCAGCAGCTGGTCA
TCGTGATGGATCCATCGGGCAACCGCCACGAGATCGTCTGGGGCTTCAAGTCCGACTTC
ACTCACTTTGCCTCGCCCCAAGGGGTGTCGCGCTTCATCACCGGCGACTACGGCCTGGG
CCATACCGTGCTGCCGGCGCCCGAGTTCGACAAGACCGTGGCTTTTGCGCGCGACGTGC
TGGGTTTCGACCTTTCTGACATCTACAACTTCAAGCCTGCGGGCGATGCCGGTCCCACG
ATCCGCATCCACTTCTTCCACTGCGCCAACGGCCGCCACCACAGCCTGGCGCTGGCCGA
GTTCCCTTCTGCATCGGGCTGCGTGCACGTGATGGTGGAGGTGGACAACATGCCCGAGG
TGGGCCGCGCCATGGACCGCATGCAAAAGAGCCAGGTCAAGCTGTCCGCCACCCTGGG
CCAGCACACCAACGACAAGATGATTTCGTTCTACATGAAGACGCCCTCGAACTTCGATC
TGGAGTTCGGCTATGGCGGCGCCATCGTCGACTGGGATCAGCACATCACGCATGAGTTC
ACGACCGTAAGCCTC

>Aerobic DHSTP *tesB*_36
GGTGGCCAACAAGGAAGCCTTCGAGCAGGCGCTGAAGGTGCTGCAGGCTGCCGACGT
GCACTACGAGATGGGCAGCCCCGAGCTATGCGCCAAGCGCCATGTGCAGCAGCTGGTCA
TCGTGATGGATCCATCGGGCAACCGCCACGAGATCGTCTGGGGCTTCAAGTCCGACTTC
ACTCACTTTGCCTCGCCCCAAGGGGTGTCGCGCTTCATCACCGGCGACTACGGCCTGGG
CCATACCGTGCTGCCGGCGCCCGAGTTCGACAAGACCGTGGCTTTTGCGCGCGACGTGC
TGGGTTTCGACCTTTCTGACATCTACAACTTCAAGCCTGCGGGCGATGCCGGTCCCACG
ATCCGCATCCACTTCTTCCACTGCGCCAACGGCCGCCACCACAGCCTGGCGCTGGCCGA
GTTCCCTTCTGCATCGGGCTGCGTGCACGTGATGGTGGAGGTGGACAACATGCCCGAGG
TGGGCCGCGCCATGGACCGCATGCAAAAGAGCCAGGTCAAGCTGTCCGCCACCCTGGG
CCAGCACACCAACGACAAGATGATTTCGTTCTACATGAAGACGCCCTCGAACTTCGATC
TGGAGTTCGGCTATGGCGGCGCCATCGTCGACTGGGATCAGCACATCACGCATGAGTTC
ACGACCGTAAGCCTC

>Aerobic DHSTP *tesB*_37
GGTGGCCAACAAGCAAGCCTTCGAGCAGGCGCTCGAGGTGTTGCAGGCCGCTGATGTG
CACTACGAAATGGGCAGCCCCGAGCTGTGTGCCAAGCGCCATGTGCAGGAGTTGGCCAT
CGTGACGGATCCATCCGGAAACCGTCATGAGATCGTCTGGGGCTTCAAGTCCGACTTCA
GCCATTTCGCATCGCCCCAGGGCGTGTCGCGCTTCATCACCGGTGACTATGGTCTGGGCC
ACACCGTGCTGCCTGCTCCCGACTTTGACAAGACCGTGGCCTTTGTGCGCAATGTGCTG
GGCTTTGGCCTCTCGGATATTTACAACTTCAAGCCCGCAGGCGATGCCGGCCCCACGGT
CCGAATCCACTTCTTCCACTGCGCCAACGCTCGTCACCACAGCCTGGCGCTGGCCGAAT
TCCCTTCTCCCTCGGGTTGCGTGCACGTGATGGTGGAGGTGGACAACATGCCCGAAGTG
GGCCGCGCCATGGACCGCATGCAAAAGAGCCAGGTCAAGCTCTCTGCCACTCTCGGTC
AGCACACCAACGACAAGATGATCTCGTTCTACATGAAGACGCCCTCCAACTTCGACCTG
GAGTTCGGCTATGGCGGCGCCATCATCGACTGGGAACACCACATCACGCACGAGTTCAC
GACCGTAAGCCTC

>Aerobic DHSTP *tesB*_38
GGTGGCCAACAAGCAAGCCTTCGAGCAGGCGCTCGAGGTGTTGCAGGCCGCTGATGTG
CACTACGAAATGGGCCGCCCCGAGCTGTGTGCCAAGCGCCATGTGCAGGAGTTGGCCAT
CGTGATGGATCCGTCCGGAAACCGTCATGAGATCGTCTGGGGCTTCAAGTCCGACTTCA
GCCATTTCGCATCGCCCCAGGGCGTGTCGCGCTTCATCACCGGTGACTATGGTCTGGGCC
ACACCGTGCTGCCTGCTCCCGACTTTGACAAGACCGTGGCCTTTGTGCGCAATGTGCTG
GGCTTTGGCCTCTCGGATATTTACAACTTCAAGCCCGCAGGCGATGCCGGCCCTACGGTC
CGAATCCACTTCTTCCACTGCGCCAACGCTCGTCACCACAGCCTGGCGCTGGCCGAATT
CCCTTCTCCCTCGGGTTGCGTGCACGTGATGGTGGAGGTGGACAACATGCCCGAAGTGG
GCCGTGCCATGGACCGCATGCAAAAGAGCCAGGTCAAGCTCTCTGCCACTCTGGGTCA
GCACACCAACGACAAGATGATCTCGTTCTACATGAAGACGCCCTCCAACTTCGACCTGG
AGTTCGGCTATGGCGGCGCCATCATCGACTGGGAACACCACATCACGCACGAGTTCACG
ACCGTAAGCCTC

>Aerobic DHSTP *tesB*_39
AGTGGCCAACAAGCAAGCCTTTGAGCAGGCGCTCAAGGTGTTGCAGGCAGCTGATGTG
CACTACGAAATGGGCAGTTCCGAACTGTGTGCCAAGCGCCATGTGCAGGAGCTGGCCAT
CGTGATGGATCCGTCCGGAAATCGTCACGAGATCGTCTGGGGCTTCAAGTCCGACTTCC
GCCATTTTGCGTCGCCCCAAGGGGTGTCGCGCTTTATCACCGGCGACTATGGTCTGGGCC
ACACCGTGCTGCCCGCTCCCGACTTTGACAAGACCGTGGCCTTTGTGCGCAATGTGCTG
GGCTTTGGTCTCTCGGATATTTACAACTTCAAGCCCGCAGGCGATGCCGGCCCCACGGT
CCGAATCCACTTCTTCCACTGCGCCAACGGGCGTCACCACAGCCTGGCGCTGGCCGAGT
TCCCTTCCGCATCGGGTTGCGTGCACGTGATGGTGGAGGTGGACAACATGCCCGAAGTG
GGCCGTGCCATGGACCGCATGCAAAAGAGCCAGGTCAAGCTCTCTGCCACCCTGGGTC
AGCACACCAACGACAAGATGATCTCGTTCTACATGAAGACGCCCTCCAACTTCGATCTG
GAGTTCGGCTATGGCGGCGCCGTCATCGACTGGGAGCACCACATCACGCACGAGTTCAC
GACCGTAAGCCTC

>Aerobic DHSTP *tesB*_40
GGTGGCCAACAAGCAAGCCTTCGAGCAGGCGCTCGAGGTGTTGCAGGCCGCTGATGTG
CACTACGAAATGGGCCGCCCCGAGCTGTGTGCCAAGCGCCATGTGCAGGAGTTGGCCAT
CGTGATGGATCCGTCCGGAAACCGTCATGAGATCGTCTGGGGCTTCAAGTCCGACTTCA
GCCATTTCGCATCGCCCCAGGGCGTGTCGCGTTTCATCACCGGTGACTATGGTCTGGGCC
ACACCGTGCTGCCTGCTCCCGACTTTGACAAGACCGTGGCCTTTGTGCGCAATGTGCTG
GGCTTTGGCCTCTCGGATATTTACAACTTCAAGCCCGCAGGCGATGCCGGCCCTACGGTC
CGAATCCACTTCTTCCACTGCGCCAACGCTCGTCACCACAGCCTGGCGCTGGCCGAATT
CCCTTCTCCCTCGGGTTGCGTACACGTGATGGTGGAGGTGGACAACATGCCCGAAGTGG
GCCGCGCCATGGACCGCATGCAAAAGAGCCAGGTCAAGCTCTCTGCCACTCTGGGTCA
GCACACCAACGACAAGATGATCTCGTTCTACATGAAGACGCCCTCCAACTTCGACCTGG
AGTTCGGCTATGGCGGCGCCATCATCGACTGGGAACACCACATCACGCACGAGTTCACG
ACCGTAAGCCTC

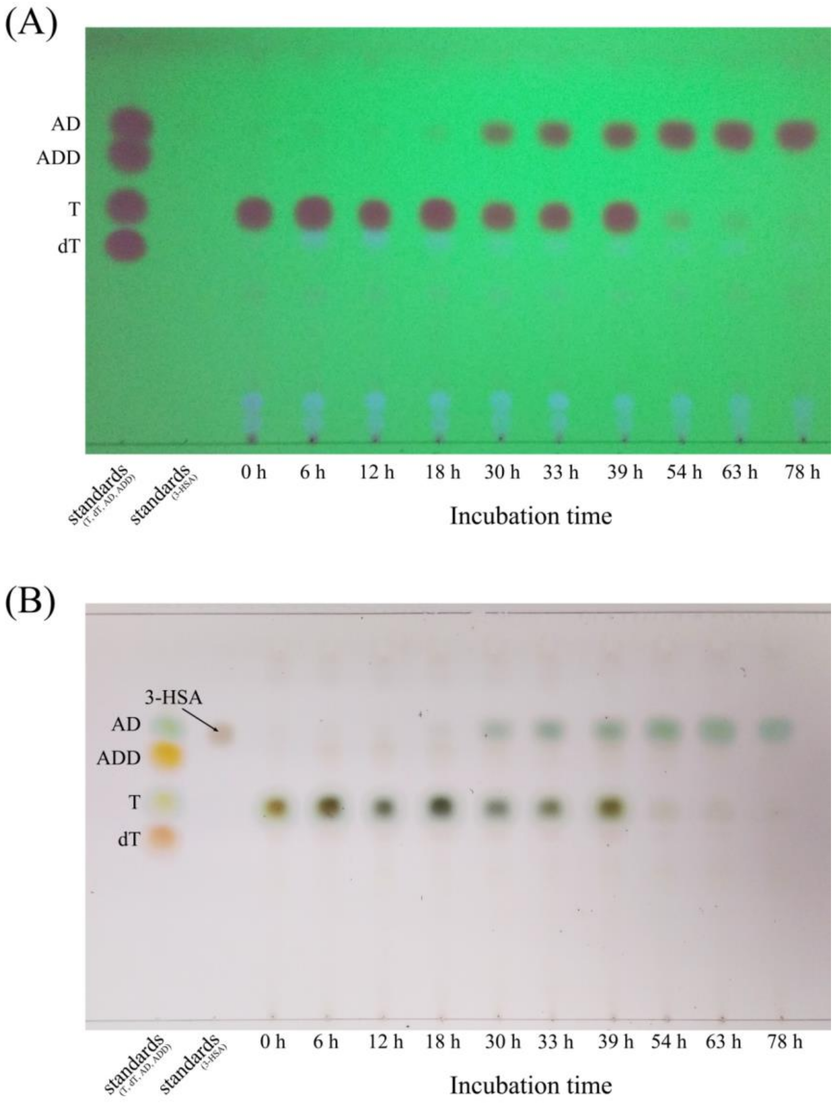

Figure S9. Thin-layer chromatograms (TLC) showing the transformation of testosterone to androst-4-en-3,17-dione by the aerobically grown *Comamonas composti* cells. *C. composti* BCRC 17659 was obtained from the Bioresource Collection and Research Center, Hsinchu, Taiwan. The bacterial culture (in 80 mL of LB medium) containing 0.5 mM testosterone was aerobically incubated at 25℃ for one week. The culture samples (1 mL) were extracted with ethyl acetate, separated by TLC, and visualized under UV light at 254 nm (top figure) or visualized by spraying the TLC plate with 30% (vol/vol) H_2SO_4 and heating (bottom image). Abbreviations: AD, androst-4-en-3,17-dione; ADD, androsta-1,4-diene-3,17-dione; dT, 1-dehydrotestosterone; T, testosterone; 3-HSA, 3-hydroxy-9,10-*seco*-androsta-1,3,5(10)-triene-9,17 -dione.

Figure S10. (A) The original full-length agarose gel for the cropped gel image shown in Fig. 5A. (B) The original full-length agarose gel for the cropped gel image shown in Fig. 5B.

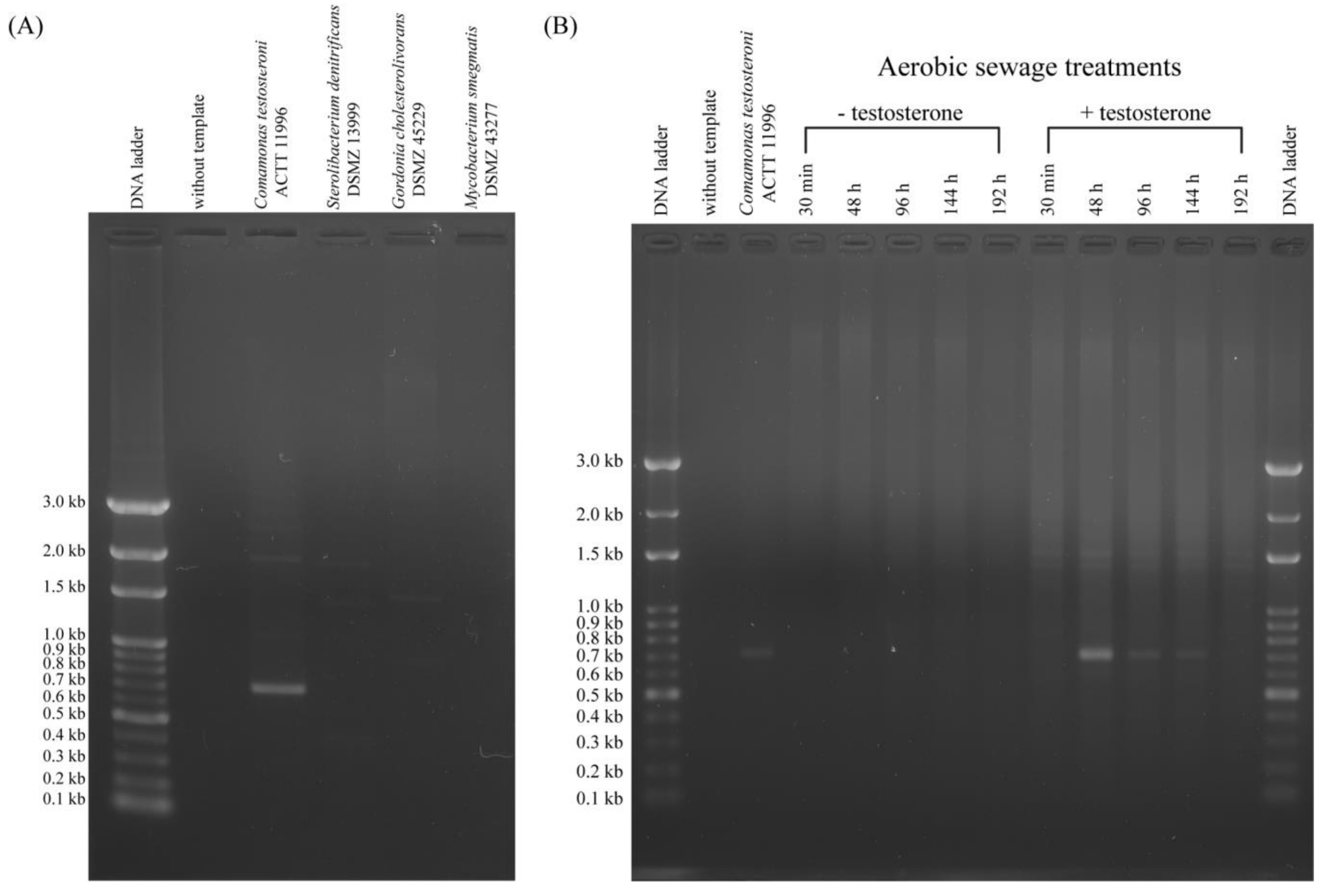

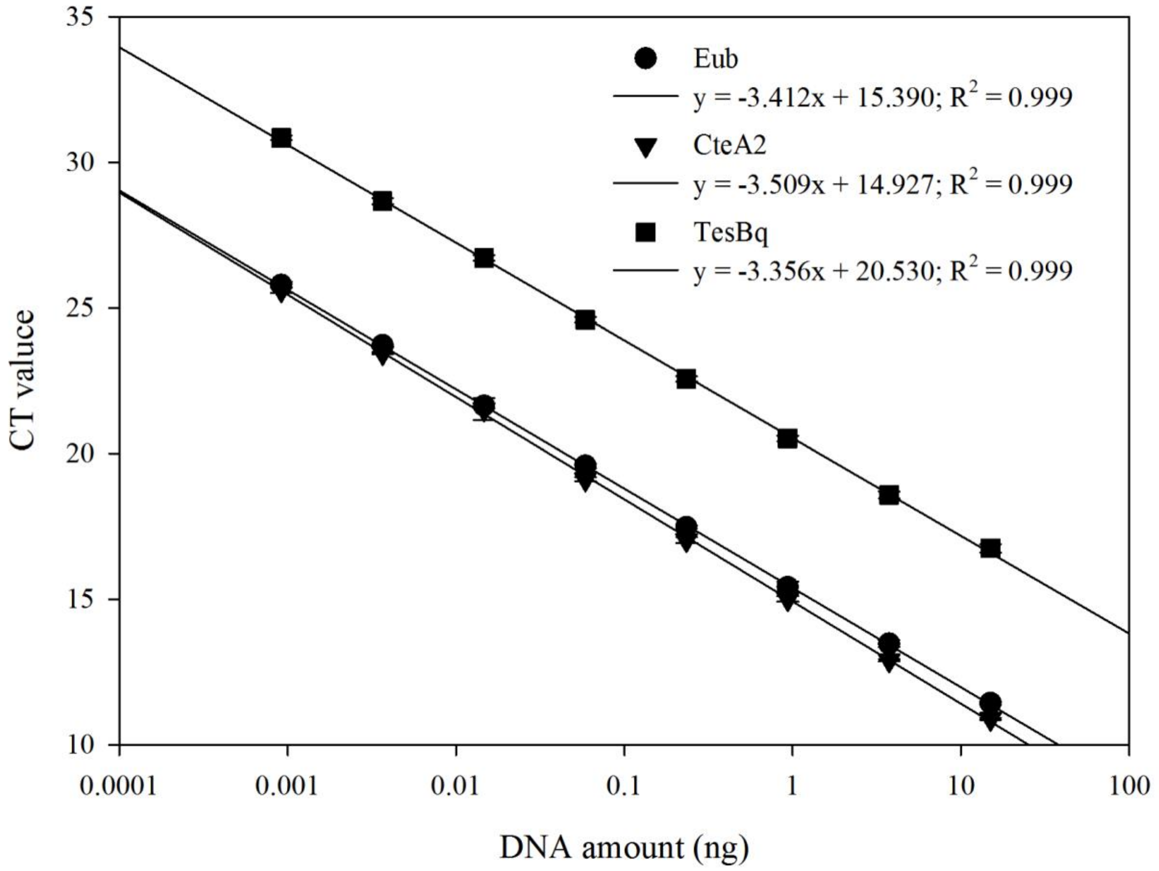

Figure S11. Real-time PCR standard curves obtained using primer pairs Eub (circles), CteA2 (triangles) and TesBq (squares) using 4 fold serial dilutions of *C. testosteroni* genomic DNA as template DNA. Linear regressions are calculated using the dots of each series.
